# Structural insights into human organic cation transporter 1 transport and inhibition

**DOI:** 10.1038/s41421-024-00664-1

**Published:** 2024-03-15

**Authors:** Shuhao Zhang, Angqi Zhu, Fang Kong, Jianan Chen, Baoliang Lan, Guodong He, Kaixuan Gao, Lili Cheng, Xiaoou Sun, Chuangye Yan, Ligong Chen, Xiangyu Liu

**Affiliations:** 1 State Key Laboratory of Membrane Biology, Tsinghua-Peking Center for Life Sciences, School of Pharmaceutical Sciences, Tsinghua University, Beijing, China; 2 Beijing Frontier Research Center for Biological Structure, Tsinghua University, Beijing, China; 3 State Key Laboratory of Membrane Biology, Tsinghua-Peking Center for Life Sciences, School of Life Sciences, Tsinghua University, Beijing, China; 4 School of Pharmaceutical Sciences, Key Laboratory of Bioorganic Phosphorus Chemistry and Chemical Biology (Ministry of Education), Tsinghua University, Beijing, China; 5 School of Basic Medicine Sciences, Tsinghua University, Beijing, China; 6 Beijing Key Laboratory of Cardiovascular Receptors Research, Peking University, Beijing, China

**Keywords:** Cryoelectron microscopy, Mechanisms of disease

## Abstract

The human organic cation transporter 1 (hOCT1), also known as SLC22A1, is integral to hepatic uptake of structurally diversified endogenous and exogenous organic cations, influencing both metabolism and drug pharmacokinetics. hOCT1 has been implicated in the therapeutic dynamics of many drugs, making interactions with hOCT1 a key consideration in novel drug development and drug–drug interactions. Notably, metformin, the frontline medication for type 2 diabetes, is a prominent hOCT1 substrate. Conversely, hOCT1 can be inhibited by agents such as spironolactone, a steroid analog inhibitor of the aldosterone receptor, necessitating a deep understanding of hOCT1–drug interactions in the development of new pharmacological treatments. Despite extensive study, specifics of hOCT1 transport and inhibition mechanisms remain elusive at the molecular level. Here, we present cryo-electron microscopy structures of the hOCT1-metformin complex in three distinct conformational states — outward open, outward occluded, and inward occluded as well as substrate-free hOCT1 in both partially and fully open states. We also present hOCT1 in complex with spironolactone in both outward and inward facing conformations. These structures provide atomic-level insights into the dynamic metformin transfer process via hOCT1 and the mechanism by which spironolactone inhibits it. Additionally, we identify a ‘YER’ motif critical for the conformational flexibility of hOCT1 and likely other SLC22 family transporters. Our findings significantly advance the understanding of hOCT1 molecular function and offer a foundational framework for the design of new therapeutic agents targeting this transporter.

## Introduction

The translocation of many drugs and endogenous molecules across the cellular and organelle membranes is facilitated by transporter proteins. Among these, the organic cation transporter OCT1 (SLC22A1), a member of the SLC22 family, is predominantly expressed in the liver and was first identified in 1994^1^. OCT1 has been characterized as a key hepatic transporter for various pharmacologically significant agents, including metformin^2–4^, the anti-cancer drug oxaliplatin^5^, and the opioid morphine^6^. OCT1 is also known to transport thiamine with high capacity, thereby regulating hepatic steatosis by modulating cellular energy status^7^, with additional roles as a neurotransmitter transporter that controls levels of circulating catecholamines^8^. The function of hOCT1 impacts the pharmacokinetics of many drugs^9^, and pharmacogenomic studies have shown genetic polymorphisms impacting hOCT1 activity can modify drug effects, sometimes resulting in adverse effects^9–13^. Although hOCT1 is the primary hepatic transporter, hOCT family members hOCT2 and hOCT3 function in renal and other organ systems^1,14–16^. Despite high sequence homology, OCT1, OCT2, and OCT3 exhibit distinct differences in tissue distribution, substrate specificity, and pharmacological inhibition, underscoring their unique physiological roles. A clear understanding of these differences is essential for the development of precision medicine strategies.

As part of the major facilitator superfamily (MFS), hOCT1 operates on an alternating access mechanism, allowing substrate-binding sites to be exposed alternately to either side of the membrane via transporter conformational changes^17^. Each hOCT1 transport cycle involves transitioning from the outward open to the outward occluded, then inward occluded, and finally the inward open states. Despite recent advances in defining the transport mechanism elucidated by examining the outward-facing conformation of hOCT3^18^, consensus mutant of hOCT1 (OCT1cs), and consensus mutant of hOCT2 (OCT2cs)^19^, the dynamic substrate translocation process remains poorly understood. To fully define the transport mechanism, detailed structural information of hOCT1 transformational states is required.

In this study, we elucidate the cryo-EM structures of hOCT1 in complex with metformin, capturing three critical conformational states, as well as substrate-free hOCT1 in an inward open state. Moreover, we present the structure of hOCT1 bound to the inhibitor spironolactone, revealing insights into the molecular mechanisms of transport and inhibition by hOCT1.

## Results

### Structure determination of hOCT1

hOCT1 was expressed in *Spodoptera frugiperda (Sf9)* insect cells and purified with high purity, following optimized purification protocols detailed in the “Materials and methods” section (Supplementary Fig. S1a–d). As a member of the MFS transporters, hOCT1 is presumed to utilize an alternating access mechanism for substrate transport. However, conformational heterogeneity inherent to this mechanism of transport poses challenges for structural determination. To address this, we sought to stabilize hOCT1 in various conformations by modifying the sample preparation conditions. These modifications included using different inhibitors, detergents, and reconstruction within nanodiscs containing distinct lipid compositions.

Initially, we analyzed the hOCT1-spironolactone complex in both DDM and LMNG micelles. Notably, the complex within DDM micelles revealed a distinct intracellular helix (ICH) domain feature using 2D classification, which was absent in LMNG-purified samples (Supplementary Figs. S2 and S3). This observation led us to hypothesize that hOCT1-spironolactone adopts distinct conformations dependent on the detergent environment. Consequently, cryo-EM datasets were collected for the complex using both micelle types (Supplementary Figs. S2 and S3). The hOCT1-spironolactone complex stabilized by DDM-containing micelles (hereby named hOCT1-S1) was resolved at 3.27 Å resolution, exhibiting an outward-facing conformation. Meanwhile, the LMNG-stabilized complex (hOCT1-S2) was determined at 2.98 Å resolution, exhibiting an inward facing structure (Supplementary Figs. S2 and S3).

**Fig. 1:**
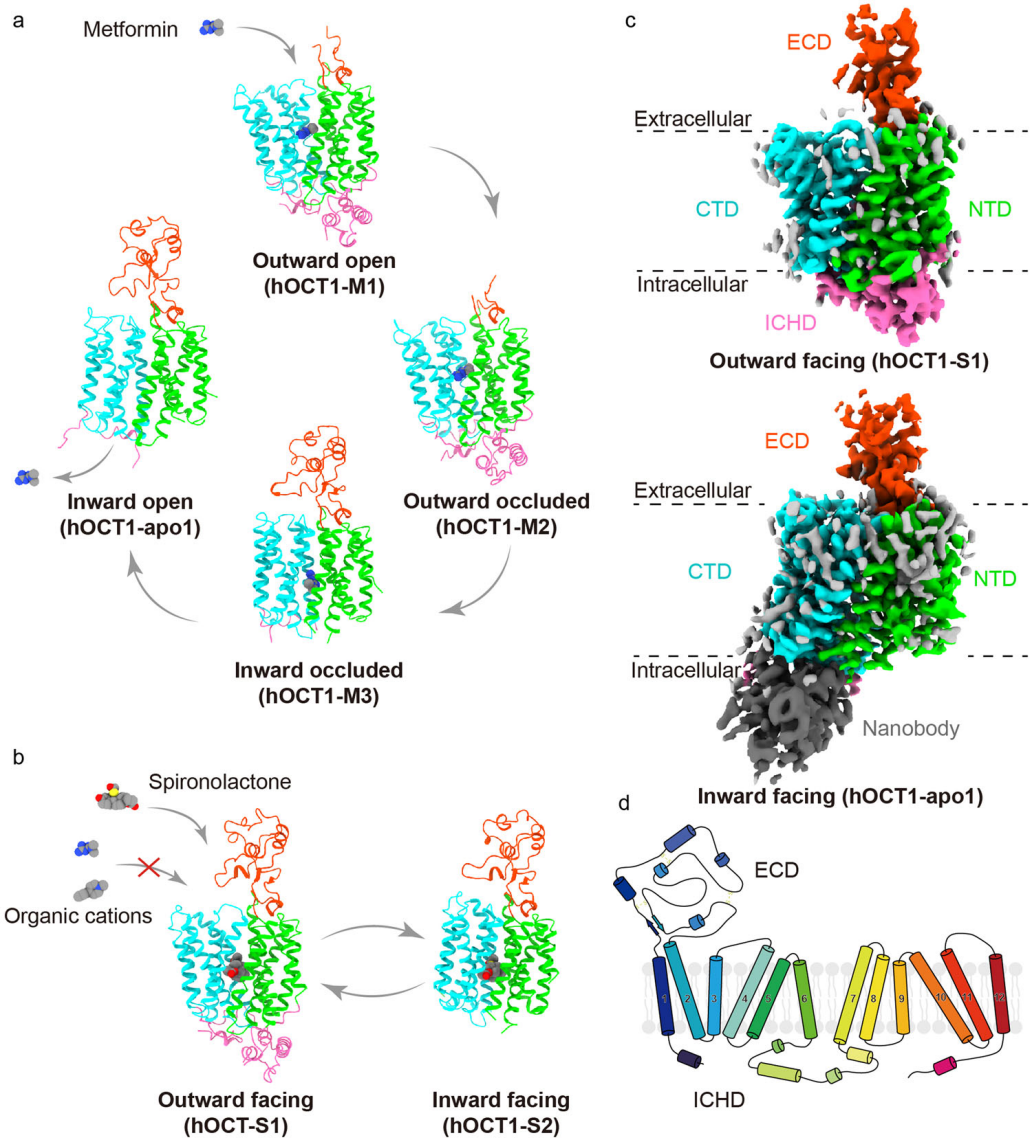
The cryo-EM structure of hOCT1 in different conformations. **a** The overall structure of hOCT1-metformin complexes in four different conformations. Metformin binds to hOCT1 in outward open (hOCT1-M1), outward occluded (hOCT1-M2) and inward occluded (hOCT1-M3) conformations. Metformin is released in the inward open (hOCT1-apo1) conformation. **b** The structure of the hOCT1–spironolactone complex in outward facing (hOCT1-S1) and inward facing (hOCT1-S2) conformations. **c** Cryo-EM reconstructions of hOCT1 in outward facing (hOCT1-S1) and inward facing (hOCT1-apo1) conformations. The NTD, CTD, and ECD are colored in green, cyan, and red, respectively. ICHD is only visible in the outward facing conformation and is colored in hot pink. The nanobody that binds to CTD in the inward facing conformation is colored in gray. **d** Topology diagram of the full-length, wild-type hOCT1 construct for cryo-EM structure determination. The extracellular loop 1 forms the extracellular domain (ECD). The intracellular helix forms the intracellular helical domain (ICHD).

In the case of hOCT1-metformin in DDM micelles, we successfully delineated structures in three conformations: outward open (hOCT1-M1), outward occluded (hOCT1-M2), and inward occluded (hOCT1-M3), at resolutions ranging from 3.8 to 4.1 Å (Fig. 1a and Supplementary Fig. S4). Attempts to determine the hOCT1-metformin structure in LMNG micelles were initially unsuccessful, likely due to the conformational flexibility and preferential orientation issues. To circumvent this, we leveraged a synthetic nanobody library in yeast^20^, using hOCT1 in LMNG as a bait. Through iterative sorting, we identified Nb56, which enhanced hOCT1 thermal stability during fluorescence size exclusion chromatography (FSEC) and co-migrated with hOCT1 during size exclusion chromatography (SEC) (Supplementary Fig. S1e, f). The cryo-EM sample of the hOCT1-Nb56 complex, however, showed a preferred orientation that obscured the side view. To bypass this, we engineered a bitopic nanobody, Nb5660, by fusing Nb56 with another nanobody (Supplementary Fig. S1g, h). Examining hOCT1-Nb5660 with cryo-EM resulted in three slightly different hOCT1-Nb5660 conformations, each inward facing, at resolutions ranging from 3.23 to 3.28 Å. In these structures, labeled hOCT1-apo1, hOCT1-apo2, and hOCT1-apo3, but no metformin densities were detected near the central cavity (“Materials and methods,” and Supplementary Fig. S5).

### Overall structure of hOCT1

hOCT1 architecture conforms to the canonical two-fold pseudo-symmetry typical of the MFS transporters. The protein can be divided into several different regions, with the N-terminal domain (NTD) comprising transmembrane helices 1–6 (TMs 1–6), and the C-terminal domain (CTD), consisting of transmembrane segments 7–12 (TMs 7-12). An extracellular domain (ECD) is delineated by a loop encompassing residues 44–142, situated between TM1 and TM2. On the intracellular side, helices form the intracellular helix (ICH) domain (Fig. 1c, d).

Across all four characterized states — outward open, outward occluded, inward occluded, and inward open — the electron density maps of the transmembrane helices are resolved well enough to enable accurate model building (Supplementary Figs. S2–S5). This clarity is further enhanced by the predictive modeling of AlphaFold2^21^. However, the densities corresponding to the ICH and ECD are not uniformly defined across these states. Consistent with the findings from 2D classification, the ICH domain densities are exclusive to outward facing conformations, irrespective of hOCT1 complex formation with either metformin or spironolactone (Supplementary Figs. S2–S5). The absence of ICH domain densities in inward facing states suggests that this region remains flexible in such conformations. Conversely, the ECD exhibits more consistent densities in inward conformations, suggesting increased flexibility in outward-facing states. While clear nb56 densities are observed in the inward-open structure, the fused nanobody could not be visualized, likely due to disorder (Fig. 1c). Consistently, Nb56 binds to the CTD of hOCT1, stabilizing the transporter in the inward open state (Supplementary Fig. S6a).

### Alternating access enables metformin translocation

**Fig. 2:**
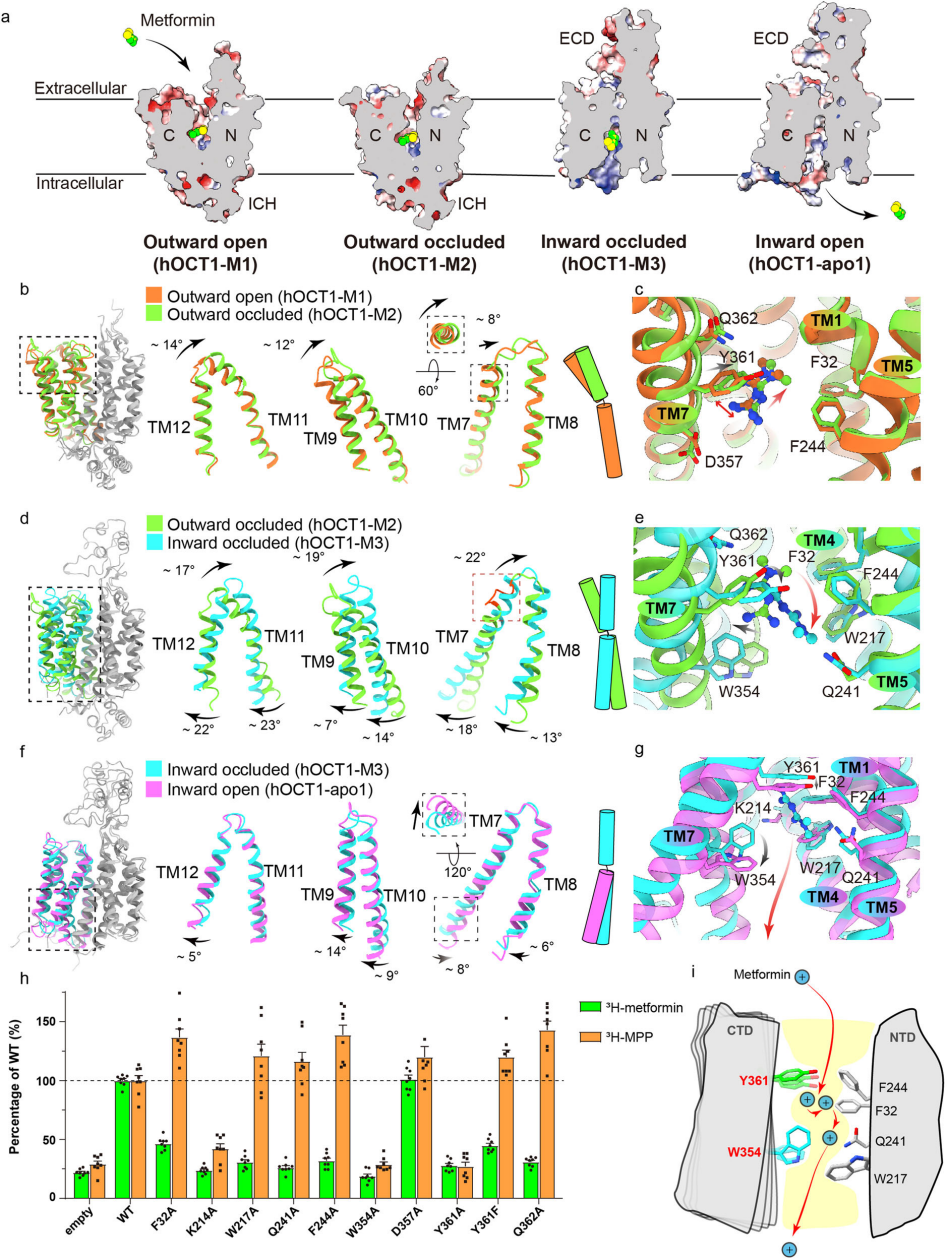
Alternating access enables metformin translocation. **a** A sliced view of the hOCT1 in the outward open (hOCT1-M1), outward occluded (hOCT1-M2), inward occluded (hOCT1-M3), and inward open (hOCT1-apo1) conformations. Metformin binds to the central pocket of hOCT1 in different states. The surface of the hOCT1 is colored according to the electrostatic potential calculated by ChimeraX. **b** Structural comparison of the outward open (hOCT1-M1) (orange) and outward occluded (hOCT1-M2) (green) conformations. The extracellular ends of transmembrane helices in the CTD (black dashed box) exhibit conformational rearrangement. **c** Structural comparison of the metformin binding pockets in the outward open (hOCT1-M1) (orange) and outward occluded (hOCT1-M2) (green) conformations. Compared to the outward open conformation, metformin moves slightly towards the NTD in the outward occluded conformation. A red double arrow shows the cation-π interaction between Y361 and metformin. **d** Structural comparison of the outward occluded (hOCT1-M2) (green) and inward occluded (hOCT1-M3) (cyan) conformations. The transmembrane helices in the CTD (black dashed box) exhibit conformational rearrangements. The red dashed box highlights the extracellular end of TM7 that rearranges from a loop to a helix during conformational change. **e** Structural comparison of the metformin binding pockets in outward occluded (hOCT1-M2) (green) and inward occluded (hOCT1-M3) (cyan) conformations. Metformin moves to a deeper position in the inward occluded conformation. **f** Structural comparison of the inward occluded (hOCT1-M3) (cyan) and inward open (hOCT1-apo1) (magenta) conformations. The intracellular ends of transmembrane helices in the CTD (black dashed box) exhibit conformational rearrangement. **g** Structural comparison of the metformin binding pockets in inward occluded (hOCT1-M3) (cyan) and inward open (hOCT1-apo1) (magenta) conformations. **h** Mutating the residues at the metformin binding pocket resulted in varied effects on the uptake activity of metformin (green) and MPP^+^ (orange). Data are normalized to wild-type hOCT1 and are given as mean ± SEM of four independent samples. **i** During the metformin translocation cycle, the CTD of hOCT1 undergoes notable conformational rearrangement. The residues Y361 and W354 play key roles in the process.

We elucidated the structures of hOCT1 in complex with metformin across three conformations: outward open (hOCT1-M1), outward occluded (hOCT1-M2) and inward occluded (hOCT1-M3) (Supplementary Fig. S6b–d), as well as unbound hOCT1 in an inward open conformation (hOCT1-apo1-3) (Supplementary Fig. S6e). These structures provide a window into the dynamic translocation of metformin via an alternating access mechanism (Fig. 2a).

Through these state transitions, the NTD of the hOCT1 remains relatively stable, with an RMSD of 1.4 Å for Cα atoms between the outward open and inward open conformations. In contrast, the CTD exhibits significant conformational shifts, reflected by an RMSD of 6.5 Å for Cα atoms between the different states (Supplementary Fig. S7a).

In its outward open state, the hOCT1 metformin-binding site exposed extracellularly. The channel connecting the extracellular environment to the binding site is negatively charged, potentially enhancing selectivity for positively charged organic cation substrates (Fig. 2a). Metformin is situated within the central cavity, forming cation-π interaction with Y361 on TM7, with Q362 also likely contributing to substrate binding (Fig. 2c). The shift from the outward open to the occluded state is characterized by the movement of the extracellular termini of the CTD helices, with the intracellular portions of these helices exhibiting minimal displacement (Fig. 2b). This results in channel narrowing, as helices TM9, TM10, TM11, TM12 converge towards the cavity, followed by TM7 approaching the central cavity by moving ~ 1.5 Å (Fig. 2b and Supplementary Fig. S7b). These conformational changes promote the slightly inward shift of Y361, narrowing the extracellular channel and causing a slight movement of metformin in the direction of the NTD (Fig. 2b).

During transition from the outward occluded state to the inward occluded state, both the extracellular and intracellular ends of the CTD helices rearrange significantly (Fig. 2d and Supplementary Fig. S7c). This realignment results in the extracellular end of TM7 tilting inward and the intracellular end shifting outward, effectively sealing the central cavity externally while opening it internally, facilitating the switch from an outward to an inward facing state (Fig. 2d). Subsequently, TM7 undergoes additional inward movement, with extracellular end transitioning from a loop to a helical structure and pushing metformin closer to an aromatic bundle of residues composed of F32, F244, and W217 from the NTD (Fig. 2e). Mutating these aromatic residues inhibited metformin transport by hOCT1 (Fig. 2h), demonstrating the importance of maintaining the cation–π interaction between Y361 and metformin. During the transition, W354 moves outwardly, contributing to the opening of the intracellular channel. This process involves the disruption and formation of two pairs of salt bridges that might contribute to inward and outward molecular gating (Supplementary Fig. S7e). Mutations of residues participating in the salt bridges resulted in decreased transport activity (Supplementary Fig. S7f).

The final transition from the inward occluded state to the inward open state is marked by further movement of the intracellular ends of the CTD helices, creating a fully open channel that bridges the central cavity with the cytoplasm (Fig. 2f). The extracellular ends of these helices remain largely static during this process. W354 undergoes a side chain rotation (Fig. 2g), with mutations at this site affecting metformin transport (Fig. 2h).

As mentioned earlier, hOCT1 functions to transport a variety of diverse organic cations (Supplementary Fig. S7a), so we next checked how mutations in the channel affected the transport of two other substrates, 1-methyl-4-phenylpyridinium (MPP^+^) and 4-(4-(dimethylamino)styryl)-*N*-methylpyridinium iodide (ASP^+^). Intriguingly, mutations along the metformin translocation pathway, excluding those at K214, W354, and Y361, did not significantly affect the uptake of MPP^+^ and ASP^+^ (Fig. 2h and Supplementary Fig. S8b). This highlights the critical roles of K214, W354, and Y361 in the hOCT1 transport mechanism. K214, located on TM4, is pivotal for bridging the NTD and CTD through a salt bridge with the residue D474 located on TM11, as reported in a recent publication^19^. Y361 translocation is associated with movement of channel substrates, while W354 side chain rotation is crucial for the opening and closing of the inward channel, as discussed in greater detail below.

### Mechanism of conformation rearrangement

**Fig. 3:**
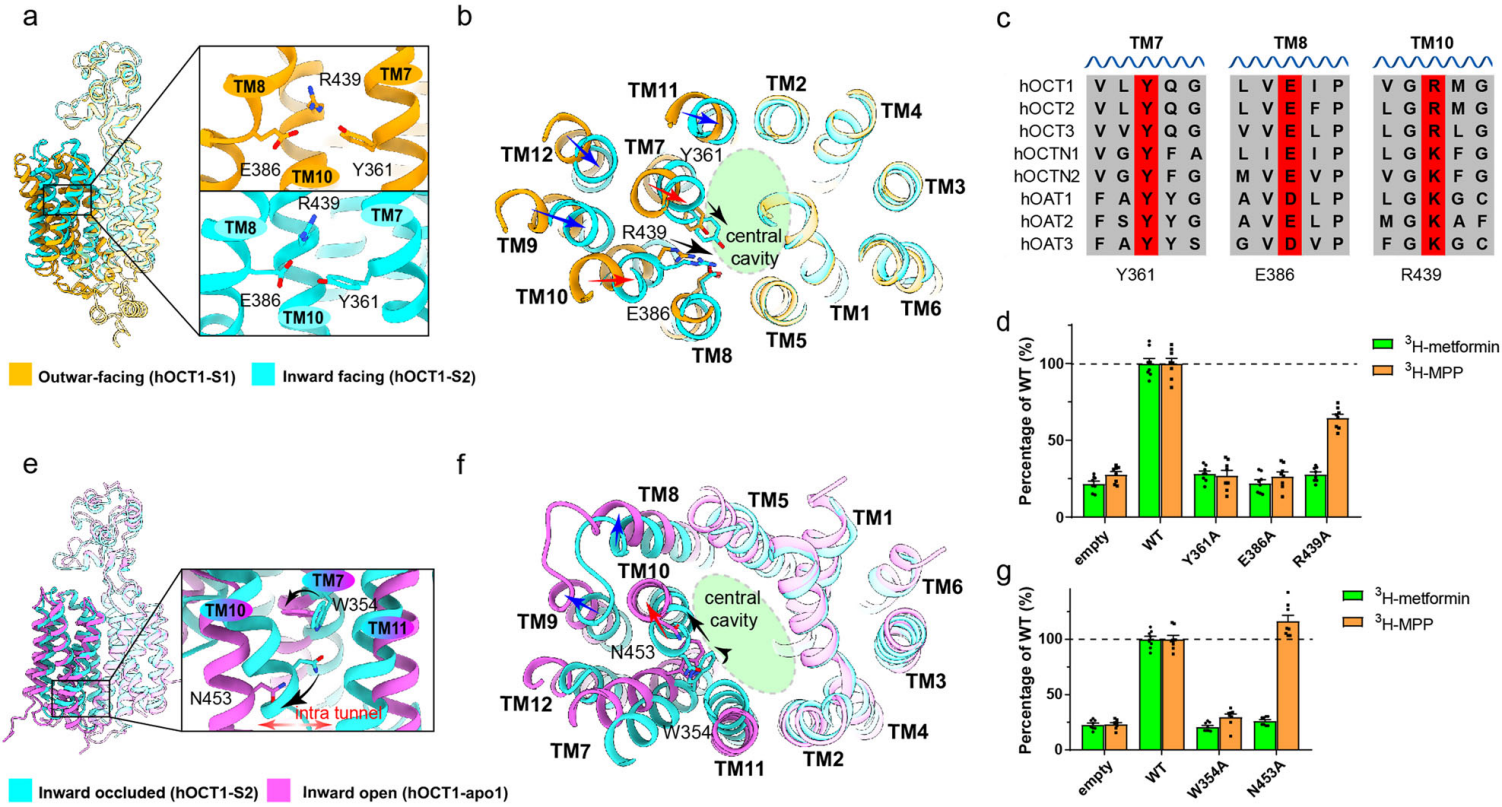
Key residues involved in the conformational change of hOCT1. **a** Overall structures of outward facing (hOCT1-S1) (orange) and inward facing (hOCT1-S2) (cyan) hOCT1 with a close-up view of the YER motif. **b** Top view of hOCT1 in the outward facing (hOCT1-S1) (orange) and inward facing (hOCT1-S2) (cyan) conformations reveals that the YER motif links change in the central pocket during the conformational rearrangement of the CTD. Black arrows indicate YER motif side chain rearrangements. Red arrows indicate movement of TM7 and TM10 that directly interact with the YER motif. Blue arrows indicate the movement of TM9, TM11 and TM12 that are indirectly connected to YER motif. **c** Sequence alignment suggests that the YER motif (highlighted in red) is conserved in SLC22A1-8. **d** Mutating any YER motif residue impaired metformin (green) and MPP^+^ (orange) uptake activities of hOCT1. Data are normalized to wild-type hOCT1 and are shown as mean ± SEM of four independent experiments. **e** W354 interacts with N453 in the inward occluded (hOCT1-S2) (cyan) state, while the interaction is broken by the inward open (hOCT1-apo1) (magenta) state. **f** Bottom view of hOCT1 in the inward occluded (hOCT1-S2) (cyan) and inward open (hOCT1-apo1) (magenta) states. The side chain rotation of W354 and the movement of N453 are associated with the inward channel opening. Black arrows indicate the movement of W354 and N453. Red arrow indicates the displacement of TM10 that directly links with N453. Blue arrows indicate the outward displacement of TM8 and TM11 that is influenced by TM10. **g** The W354A mutation impairs metformin (green) and MPP^+^ (orange) uptake by hOCT1, while the N453A mutation only impairs the metformin (green), but not the MPP^+^ (orange), uptake activity of hOCT1. Data are normalized to wild-type hOCT1 and are shown as mean ± SEM of four independent experiments.

Structural analysis and complementary mutagenesis results identified Y361 as a pivotal component in the hOCT mediated transport of organic cation substrates. To understand how the conformation of Y361 changes in various states, we examined neighboring residues. Intriguingly, residues E386 from TM8 and R439 from TM10 were shown to closely interact with Y361 to form a polar interaction network, which was maintained in both outward facing and inward facing conformations with minor variation of interactions (Fig. 3a and Supplementary Fig. S9a). This interaction network functions to link TM7, TM8, and TM10, playing a crucial role during substrate translocation. Substrate translocation involves the inward movement of Y361, which is accompanied by the inward movement of R439 (Fig. 3b). Thus, TM7 and TM10 pivot towards the central cavity, prompting alterations within the central pocket. The movement of TM7 and TM10 is synchronized with the movement of the adjacent TM9, TM11, and TM12, which interact extensively with TM7 and TM10 within the transmembrane domain (Supplementary Fig. S7c). Thus, the Y361–E386–R439 interaction is instrumental in coupling substrate translocation with conformation rearrangements of the hOCT1.

These interactions underscore the conservation of a critical motif across the SLC22 family, which encompasses OCTs, OCTNs, and OATs (Fig. 3c). The motif consists of a strictly conserved tyrosine (Y) on TM7, a negatively charged residue (either glutamate, E or aspartate, D) on TM8, and a positively charged residue (either arginine, R, or lysine, K) on TM10, together forming the “Y-E/D-R/K” motif (Supplementary Fig. S9b–f). “YER” motif integrity is critical, and mutations in any of these residues leads to impaired transporter function (Fig. 3d and Supplementary Fig. S9g).

At the intracellular juncture of TM7 and TM10, W354 forms a critical interaction with N453 when hOCT1 is in the inward occluded conformation. This interaction is absent in the inward open conformation, where W354 is seen to undergo a significant side chain rotation (Fig. 3e). This structural shift is essential for the ~4 Å outward movement of TM7 and ~6 Å outward movement of TM10, which enlarges the inward channel (Fig. 3e, f). Mutagenesis experiments supported the importance of this interaction in metformin translocation (Fig. 3g and Supplementary Fig. S9h).

### Gene polymorphism affects hOCT1 function

**Fig. 4:**
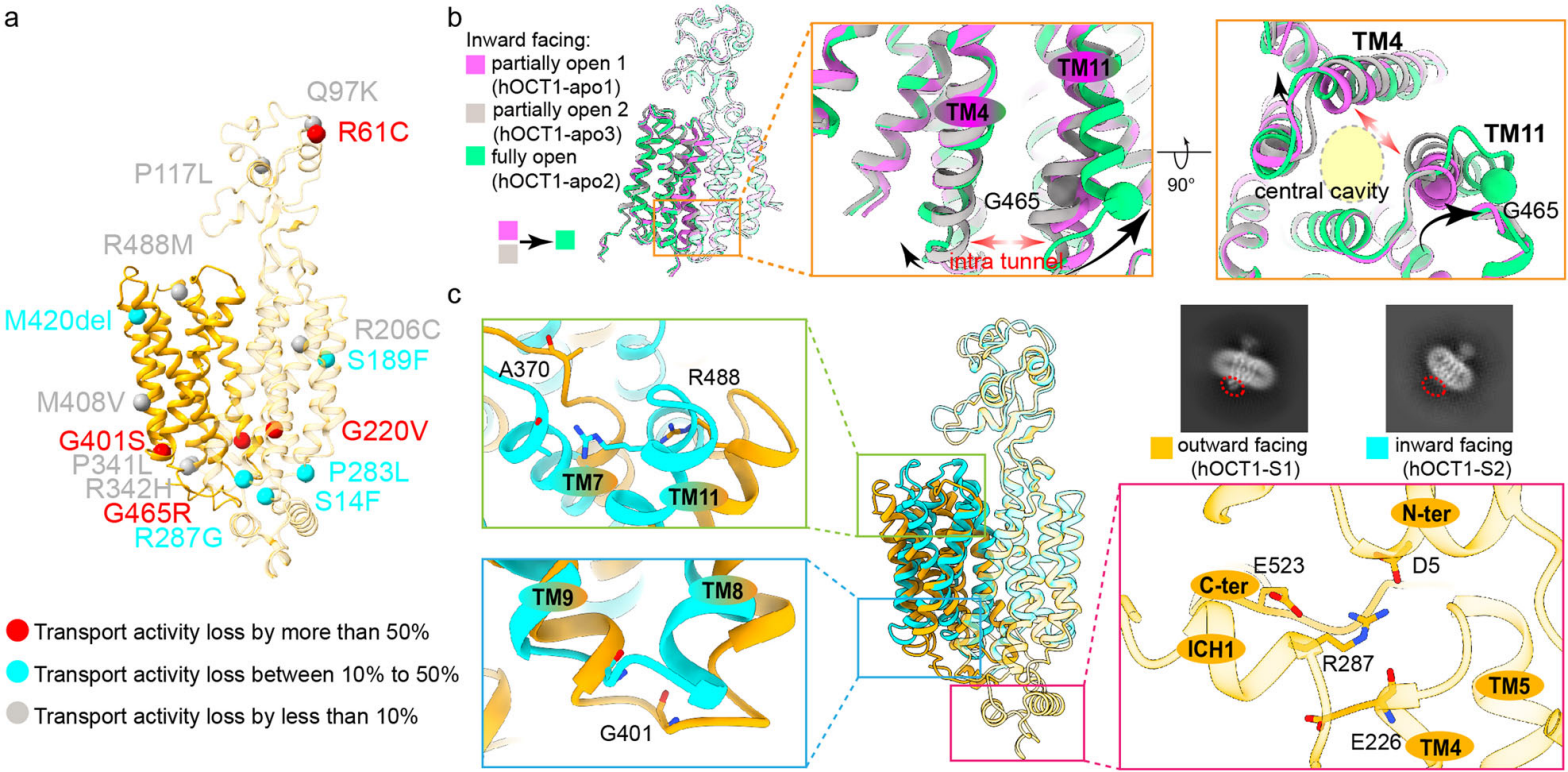
Genetic polymorphisms related to conformational changes of hOCT1. **a** Genetic polymorphisms identified in hOCT1 shown on the structure. Mutations are shown as spheres and grouped into three categories: impaired transport activity by more than 50% (red), impaired transport activity by 10% to 50% (cyan), and impaired transport activity by less than 10% (dark gray). **b** Structural comparison of the inward facing partially open 1 (hOCT1-apo1) (magenta), partially open 2 (hOCT1-apo3) (gray), and fully open (hOCT1-apo2) (green) conformations with a close-up view of the unwinding of TM11 at G465 at the fully open conformation. G465 is shown as sphere. The partially open 1 state and partially open 2 state have different TM4 and TM11 conformations. **c** Structural comparison of the outward facing (hOCT1-S1) (orange) and inward facing (hOCT1-S2) (cyan) conformations with a close-up view of R488 (left top), G401 (left bottom), and R287 (right bottom). Representative 2D class averages (top right) of hOCT1 corresponding to outward facing and inward facing conformations suggest that the ICHD is only visible at the outward facing conformation.

Previous studies have established that genetic polymorphisms influencing OCT1 activity can modulate the pharmacokinetics of drugs that are organic cations, affecting therapeutic efficacy and the likelihood of potential adverse effects^9–13^. To determine the functional impact of these genetic variations, we mapped reported mutations on the hOCT1 structure. Interestingly, these mutations were predominantly located in the transporter’s peripheral regions, rather than the central cavity where the substrate binding occurs (Fig. 4a). These mutations had varied effects on the hOCT1 activity. In Fig. 4a, mutated residues were colored according to how they influenced transport activity: gray mutations reduced activity by less than 10%, red mutations reduced activity by more than 50%, and cyan mutations had other consequences. The majority of cyan/red mutations were clustered on the intracellular portion of the hOCT1, highlighting the importance of this region for regulating substrate transport.

High-resolution structures of hOCT1 across various conformations enabled us to analyze how some of these mutations might alter the conformation of hOCT1 and subsequently impact its function. One interesting mutation site is G465^22,23^. When examining the inward open conformation dataset, we discerned three distinct classes of conformations: two partially open and one fully open, with overall RMSD values below 0.4 Å (among Cα atoms) indicating close conformational similarity (Supplementary Fig. S5). The most pronounced divergences were detected at the intracellular ends of TM4 and TM11, with RMSD values range from 2 Å (between partially open 1 and partially open 2), to 3.7 Å (between partially open 1 and fully open) and 3.7 Å (between partially open 2 and fully open). Notably, in the fully open state, TM11 exhibited a distortion at the G465 residue, promoting a helix to loop transition facilitating the opening of the intracellular side of the central channel to facilitate substrate release (Fig. 4b). The G465R mutation was shown to significantly impair transport activity^7,22,23^, possibly by altering the conformational switch between this fully open state and other states. Other mutations of interest include R488M, G401S and R287G. R488 interacts with an oxygen in the main chain of A370 in inward facing conformational states, but not in outward facing conformations due to the unwinding of the extracellular end of TM7 (Fig. 4c). The R488M mutation was shown to alter transport kinetics of hOCT1^24^. G401, another residue of interest, is located in the short loop between TM8 and TM9 that undergoes large conformational changes during the transport cycle (Fig. 4c). The flexible nature of glycine residue in this position allows the loop to adopt different conformations in different states, while substitution with serine (G401S) reduces flexibility and significantly impairs substrate transport by hOCT1^25,26^. R287 is located at the ICH domain and interacts with D5, E226 and E523 in the outward facing conformations. This interaction network serves as a hub connecting the N and C termini. The interaction is absent in inward facing conformations (Fig. 4c). The R287G mutation disrupts this interaction and significantly affects the transport activity of hOCT1^27,28^.

### Mechanism of hOCT1 inhibition by spironolactone

**Fig. 5:**
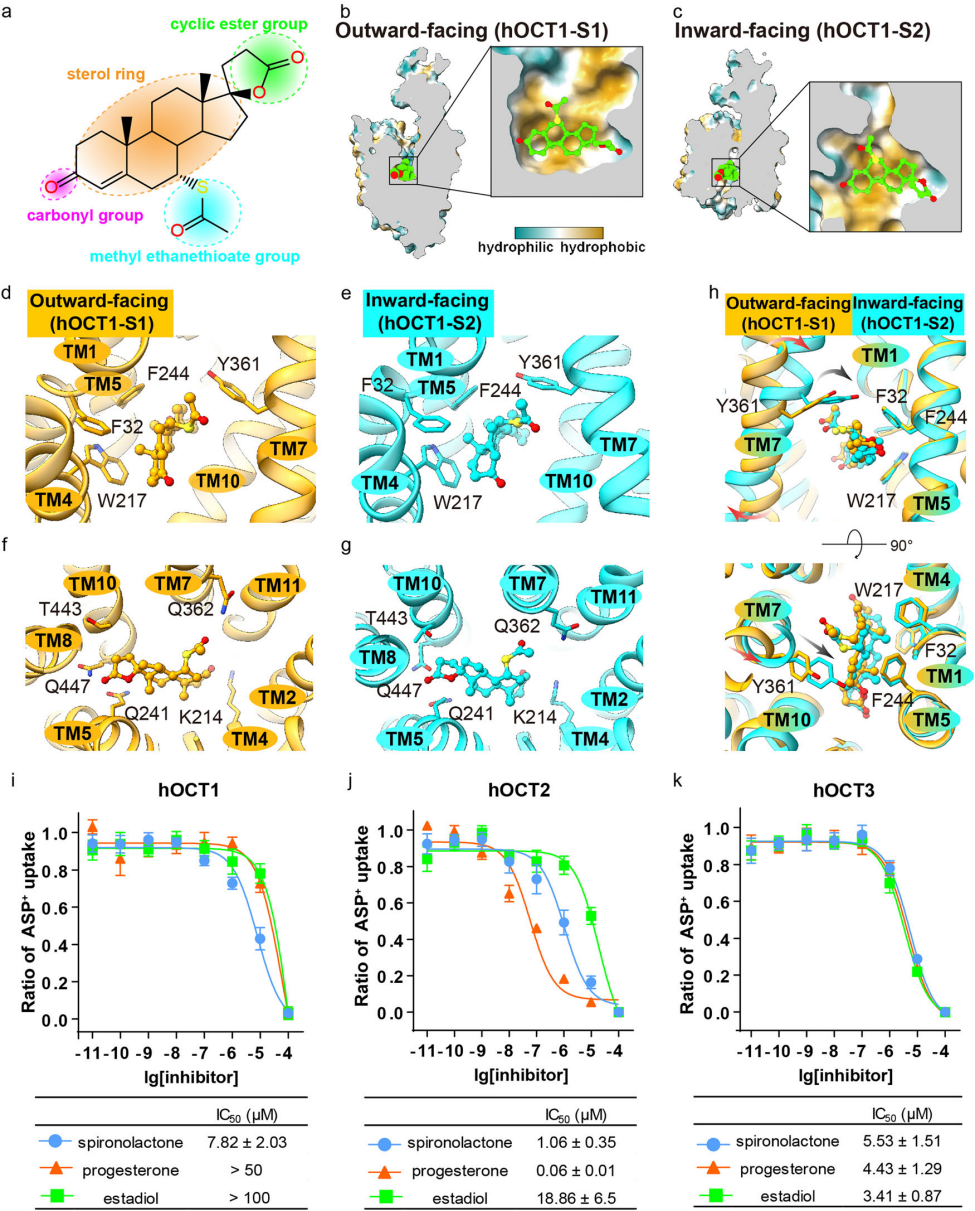
Molecular basis of hOCT1 inhibition by spironolactone. **a** The chemical structure of spironolactone. **b**, **c** Spironolactone binds to the central pocket of hOCT1 at the outward facing (hOCT1-S1) (**b**) and inward facing (hOCT1-S2) (**c**) conformations. The binding pocket is hydrophobic, as shown by the molecular lipophilicity potential function in Chimera X. **d**, **e** Hydrophobic interactions between spironolactone and hOCT1 in the outward facing (hOCT1-S1) (**d**) and inward facing (hOCT1-S2) (**e**) conformations. **f**, **g** Hydrophilic interactions between spironolactone and hOCT1 in the outward facing (hOCT1-S1) (**f**) and inward facing (hOCT1-S2) (**g**) conformations. **h** Structural comparison of the spironolactone-binding pockets in outward facing (hOCT1-S1) (orange) and inward (hOCT1-S2) (cyan) facing conformations. **i**–**k** Inhibition of ASP^+^ uptake by spironolactone (cyan), progesterone (orange), and estradiol (green) in HEK293T cells stable expressing hOCT1 (**i**), hOCT2 (**j**), and hOCT3 (**k**). Data are shown as mean ± SEM of three independent experiments.

Inhibition of hOCT1 function alters the pharmacokinetics and therapeutic efficacy of many organic cation drugs, which account for ~ 40% of all prescribed drugs^29,30^. We therefore examined the molecular mechanism of hOCT1 inhibition using several approaches. Spironolactone, a drug targeting the aldosterone receptor to treat heart failure, has been reported as a hOCT1 inhibitor^31,32^. The structure of spironolactone contains a steroid nucleus with several modifications including a cyclic ester group, a methyl ethernethioate group and a carbonyl group (Fig. 5a). Using Cryo-EM, we obtained the hOCT1-spironolactone complex structures in both outward facing and inward facing conformations at 3.27 and 2.98 Å resolutions, respectively (Fig. 1b and Supplementary Figs. S2 and S3).

In both inward and outward facing structures, spironolactone binds to a pocket within the central cavity of hOCT1. Residues at the binding site are predominantly hydrophobic, regardless of hOCT1 conformation (Fig. 5b, c). When bound to hOCT1, the steroid ring of spironolactone forms hydrophobic interactions with the aromatic residues F32, F244, W217 and Y361 (Fig. 5d, e), which we identified as being critical for metformin transport. The cyclic ester group of spironolactone resides in a hydrophilic sub-pocket composed of residues Q241 located on TM5, and T443 and Q447 located on TM10 (Fig. 5f, g). On the opposite side of the cyclic ester group, the methyl ethanethioate group of spironolactone forms a polar interaction with residue Q362 located on TM7. The carbonyl group of spironolactone interacts with K214 from TM4 (Fig. 5f, g).

As previously described, residue Y361 located on TM7 undergoes inward movement during the transition from outward facing to inward facing states. This conformational change is capable of transporting metformin, however, does not result in notable movement of spironolactone (Fig. 5h and Supplementary Fig. S10e, f). The observation that spironolactone interacts with hOCT1 in multiple conformations suggests that spironolactone is capable of associating or dissociating with the central pocket from both inside and outside the cell.

Steroid hormones, including progesterone and estradiol, have similar chemical structures with spironolactone including a steroid nucleus, but with different hydrophilic groups attached to the ring (Supplementary Fig. S11a). Consequently, while hydrophobic stacking interactions observed between hOCT1 and spironolactone are preserved between hOCT1 and progesterone or estradiol, the polar interactions are likely not conserved. We performed a functional assay, determining that both progesterone and estradiol are capable of inhibiting hOCT1 with decreased efficiency compared to spironolactone (Fig. 5i). Our observations are consistent with previous reports suggesting that steroid hormones could bind to organic cation transporters to inhibit their function^33^. The results also suggest that stacking interactions between aromatic residues have a key functional role in transporter inhibition, with differences in polar interactions determining the efficiency of hOCT1 inhibition. We further examined the inhibition of hOCT2 and hOCT3 by spironolactone and the related steroid hormones, which have similar binding pocket architecture as hOCT1 (Supplementary Fig. S11b–d). Consistently, all three compounds (spironolactone, progesterone and estradiol) inhibited hOCT2 and hOCT3 substrate transport (Fig. 5g, h). Interestingly, progesterone was the strongest hOCT2 inhibitor (Fig. 5g). The residues C36 and I446 found in the top of the binding pocket might contribute to the different inhibition potencies (Supplementary Fig. S11b, f). We found that mutating these residues to aromatic residues results in increased inhibition of hOCT1 by progesterone (Supplementary Fig. S11g–k). The results also highlight the importance of accounting for high concentrations of progesterone or estradiol in the plasma when designing treatment strategies.

## Discussion

hOCT1-facilitated transmembrane transport of organic cations plays a pivotal role in various biological processes. Though generally accepted that MFS family transporters function through an alternating access mechanism, detailed structural information of an MFS transporter assuming different conformational states had not been reported. Recently, several structures of SLC22 family transporters were described, including OCT1, OCT2, OCT3, and OAT1^18,19,34–36^, but these studies stopped short of capturing each of the conformational states exhibited by the transporter during substrate import. In our study, we determined that hOCT1 adopts different conformations in DDM or LMNG micelles. This observation enabled us to capture hOCT1 in four different conformational states, that constituted a full cycle of the alternating access import mechanism. The ability of detergent composition to affect hOCT1 conformation suggests that hOCT1 is responsive to the surrounding environment, including the lipid composition of the membrane bilayer. This phenomenon has been observed previously for other membrane proteins, including transporters, ion channels, and GPCRs^37–39^.

**Fig. 6:**
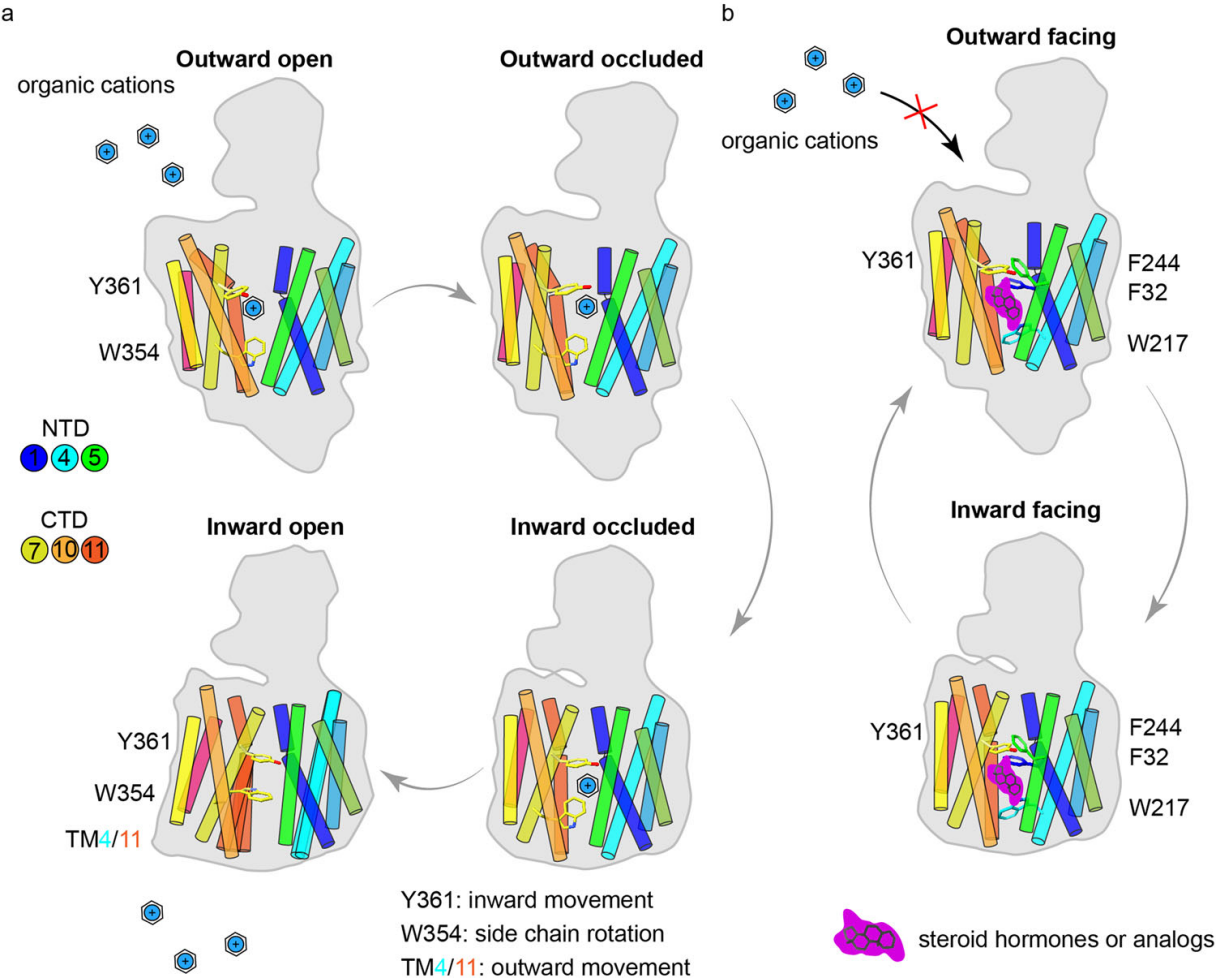
The working mechanism of hOCT1. **a** The alternating access mechanism of hOCT1-mediated organic cation transport. The organic cations (blue) bind to the central cavity of hOCT1. Y361 and W354 are involved in substrate recognition and translocation. **b** The inhibitory mechanism of steroid hormones and analogs on hOCT1. Steroid hormones or analogs (purple) occupy the central cavity of hOCT1 in outward and inward facing conformations.

High-resolution structures of the hOCT1-metformin complex in different conformations allowed the visualization of the alternating access mechanism. During the process, the NTD of hOCT1 remained relatively static, while conformational displacements of the CTD facilitated alternating access to the central pocket. The aromatic residues Y361 and W354 of the CTD played essential roles in mediating substrate binding and transport (Fig. 6a). In contrast to substrates, steroid drug inhibitors exhibit stronger interactions with aromatic residues clustered in the NTD (Fig. 6b). While this study highlighted the architecture of hOCT1 in different structural conformations, more detailed interpretations are limited by the resolution of the cryo-EM approach. Nevertheless, the results we obtained for the binding sites of metformin and spironolactone are supported by recently reported structures of the hOCT1–metformin complex in an inward facing conformation and the hOCT3–corticosterone (an analog of spironolactone) complex in an outward conformation^18,34^.

One intriguing feature of hOCT1 is that the central pocket is mainly composed of aromatic and hydrophobic residues. Previous studies have shown these residues to be essential for substrate recognition^19,40,41^. Here, we show that mutating residues within the central cavity alters the transport capacity of metformin, MPP^+^ or ASP^+^. For example, the W217A, Q241A, F244A, Y361F, and Q362A mutations strongly inhibit metformin transport, but have smaller effects on MPP^+^ transport (Fig. 2h). This result highlights the versatility of hOCT1 function. As common substrates of hOCT1, metformin, MPP^+^ and ASP^+^ each contain a positively charged cation group, but differ in terms of hydrophobicity, molecular shape and size (Supplementary Fig. S8a). Metformin is a small, hydrophilic compound, while MPP^+^ and ASP^+^ are hydrophobic compounds with aromatic rings. Our structures and the structure reported previously^19^ reveal that metformin and MPP^+^ interact differently with the substrate-binding pocket of hOCT1. For example, interactions between metformin and the aromatic bundle residues (F32, W217, F244) are mainly through cation–π interactions, while MPP^+^ interacts with these residues through hydrophobic interactions (Supplementary Fig. S8b–e). Differences in interactions may account for the variable responses to mutations in residues of the central cavity.

In our work, we found the transporter to be surrounded by a bundle of unmodelled densities, most likely derived from the alkyl chains of the surrounding detergents or lipids. Interestingly, a blob of strong electron density with an alternative shape was observed near TM2 and TM11 in the inward facing hOCT1-spironolactone complex (Supplementary Fig. S12a, b). Similar lipid-like densities have been previously observed at similar regions of hOCT3 and OATP1B1^18,42^. Together, these observations support the hypothesis that this region might act as a potential access route to the central cavity for hydrophobic substrates (Supplementary Fig. S12a, b). Another OCT feature is the low-affinity and high-capacity “uptake 2” functionality, which differs from the high-affinity and low-capacity “uptake 1” functionalities of transporters such as NET, SERT and DAT, of the SLC6 family^43–46^. Further investigation is required to elucidate the uptake 2 transporter function of OCT.

One known function of hOCT1 is the regulation of hepatic metabolism by functioning as a high-capacity thiamine transporter^7^. These observations suggest that inhibitors of hOCT1 could potentially function as therapeutics for treating nonalcoholic steatohepatitis. Inhibiting substrate transport is possible by either the competitive disruption of substrate-transporter binding, or by inhibiting conformational changes required for transporter function. In this study, we report high-resolution structures of the hOCT1 substrate-binding pocket in different conformations, and detail the multiple conformational changes undergone by hOCT1 during substrate transport, further determining that the “YER” motif is essential for proper hOCT1 function. The structures provided by this study offer valuable starting points for the design of hOCT1 inhibitors.

## Materials and methods

### Construct design, expression, and purification of recombinant hOCT1

hOCT1 was cloned into the pFastBac vector in frame with an N-terminal protein C tag and a C-terminal 3C protease cleavage site followed by a GFPuv fusion protein and a 6× His tag. Baculovirus was generated following the Bac-to-Bac protocol (Expression Systems). *Sf9* insect cells were infected with the baculovirus at a density of 4 million cells per mL. Cells were collected 72 h post-infection by centrifugation at 4000 rpm for 15 min at 4 °C and stored at –80 °C until purification.

For purification, cell pellets were thawed and resuspended in lysis buffer (20 mM Tris-HCl, pH 8.0, 2 mM EDTA, 1 mM benzamidine, 1 μg/mL leupeptin, 10 μg/mL DNase I). The lysate was centrifuged at 18,000 rpm for 15 min at 4 °C, and membrane pellets were solubilized in solubilization buffer (20 mM HEPES-NaOH, pH 8.0, 300 mM NaCl, 30% glycerol, 1% DDM, 0.1% CHS, 1 mM benzamidine, 1 μg/mL leupeptin) using a dounce homogenizer. The lysate was supplemented with 10 mM imidazole and incubated with an Ni-NTA resin for 2 h at 4 °C. The Ni-NTA resin was collected and washed with 10 column volumes (CV) of wash buffer I (20 mM HEPES-NaOH, pH 8.0, 300 mM NaCl, 30% glycerol, 0.1% DDM, 0.01% CHS, 20 mM Imidazole) and 10 CV of wash buffer II (20 mM HEPES-NaOH, pH 8.0, 300 mM NaCl, 10% glycerol, 0.1% DDM, 0.01% CHS, 20 mM Imidazole). Protein was eluted using wash buffer II plus 200 mM Imidazole. The eluted protein was supplemented with 2 mM CaCl_2_ and loaded onto a protein C column. After washing with 10 CV of wash buffer (20 mM HEPES-NaOH, pH 8.0, 300 mM NaCl, 10% glycerol, 0.1% DDM, 0.01% CHS, 2 mM CaCl_2_), the protein was eluted with elution buffer (20 mM HEPES-NaOH, pH 8.0, 300 mM NaCl, 10% glycerol, 0.02% DDM, 0.002% CHS, 5 mM EDTA, 0.2 mg/mL protein C peptide). The protein was then concentrated with 50 kDa centrifugal filter and loaded onto Superdex 200 Increase 10/300 GL (Cytiva) in the presence of size exclusion chromatography (SEC) buffer (20 mM HEPES-NaOH, pH 8.0, 100 mM NaCl, 0.02% DDM, 0.002% CHS). Peak fractions were concentrated to about 13 mg/mL and stored at –80 °C. For LMNG samples, the detergent was gradually exchanged from 0.1% DDM to 0.002% LMNG on a protein C column, using SEC buffer containing 20 mM HEPES-NaOH, pH 8.0, 100 mM NaCl, 0.002% LMNG, 0.0002% CHS.

### Thermal stability assay

hOCT1 (1 mg/mL) was used to assess thermal stability in different salt concentration and pH by the UNcle multifunctional protein stability analysis system (Unchained Labs, USA). Samples were heated from 25 °C to 95 °C at 0.4 °C/min to detect the full spectrum fluorescence and static light scattering (SLS). The data were analyzed using UNcle Analysis software (Unchained Labs, USA, Version 4.01).

For the fluorescence size exclusion chromatography thermal stability assay (FSEC-TS), hOCT1-GFP (20 μg/mL) with or without the inclusion of different nanobodies was heated to 50 °C for 5 min and rapidly cooled on ice. Heated samples were injected into the Superdex 200 Increase 5/150 GL (Cytiva) and GFP fluorescence was detected using excitation at 488 nm and emission at 525 nm.

### Nanobody selection and purification

Nanobodies that bind to the hOCT1 were identified by yeast surface display as previously reported^20^. Briefly, the yeast that could bind to hOCT1 were enriched by 3 rounds of magnetic cell sorting (MACS) and 3 rounds of fluorescence-activated cell sorting (FACS). After purification, single clones were picked and cultured in 96-well plate, and incubated with hOCT1 protein at varying concentrations. Then the binding ability was detected by flow cytometry. Nanobodies that could bind to hOCT1 were cloned to pET26b and expressed in *Escherichia coli BL21 (DE3)*. The bitopic nanobody Nb5660 was constructed by fusing Nb56 with a nanobody that cannot bind hOCT1. Nanobody purification was performed as previously described^47^. Generally, the pellets were resuspended in SET buffer (200 mM Tris, pH 8.0, 500 mM sucrose, 0.5 mM EDTA) and rotated at 4 °C. After 1 h, 2× volume of SET/4 buffer (50 mM Tris, pH 8.0, 125 mM Sucrose, 0.125 mM EDTA) was added to the solution and stirred for 1 h at room temperature. Lysates were then centrifuged at 18000 rpm, and the supernatants were loaded on an Ni-NTA resin. After washing with high salt buffer (20 mM Tris, pH 8.0, 1 M NaCl, 20 mM Imidazole) and low salt buffer (20 mM Tris, pH 8.0, 100 mM NaCl, 20 mM Imidazole), nanobodies were eluted with elution buffer (20 mM Tris, pH 8.0, 100 mM NaCl, 200 mM Imidazole), and loaded onto a Superdex 200 Increase 10/300 GL column (Cytiva) in 20 mM HEPES-NaOH, pH 8.0, 100 mM NaCl. The purified nanobodies were frozen at –80 °C for further use.

### Cryo-EM sample preparation and data collection

Purified hOCT1 in DDM (13 mg/mL) or LMNG (10 mg/mL) micelles were incubated with substrate metformin at 100 mM or the inhibitor spironolactone at 0.5× saturated concentration for 1 h at 4 °C. For hOCT1-DDM samples, 4 μL of purified protein was applied to glow-discharged (25 mA for 45 s) cryo-EM grids (Quantifoil NiTi 1.2/1.3 300 mesh). The blotted grids (5 s, 8 °C, 100% humidity) were rapidly frozen in liquid ethane cooled by liquid nitrogen with Vitrobot (FEI Mark IV, Thermo Fisher Scientific). For hOCT1-LMNG samples, a 4 μL aliquot of purified protein was applied to glow-discharged (25 mA for 30 s) cryo-EM grids (Quantifoil Au 1.2/1.3 200 mesh) and plunge-frozen with Vitrobot (4 s, 8 °C, 100% humidity). The grids were transferred to a 300 kV Titan Krios equipped with Gatan K3 Summit detector or Falcon 4 electron detector with GIF Quantum energy filter (slit width 20 eV). Micrographs of hOCT1-Nb5660-metformin were recorded with a defocus range of –1.1 to –1.5 μm and a pixel size of 0.86 Å. The total dose was about 50 e^–^/Å^2^ for each stack. For the other samples, data collection was done using EPU data acquisition software in super resolution mode with a calibrated pixel size of 0.54 Å and a defocus range of –1.1 to –1.5 μm. All 32 frames in each stack were aligned and summed using the MotionCor2 and binned to a pixel size of 1.08 Å^48^.

### Data processing

The following processes were performed using cryoSPARC 3.2 and 4.1^49^ unless otherwise specified. Dose-weighted micrographs were used for CTF estimation with patch CTF. Micrographs showing worse than 4 Å estimated CTF resolution were excluded during curating exposures. A subset of about 300 micrographs were used for blob picking of the initial particles, which were used for 2D classification to generate templates for template picking.

For hOCT1–spironolactone complexes in DDM micelles, 3,514,899 particles from 2104 micrographs were extracted with a box size of 200 pixels and cropped into 128 pixels. After three rounds of 2D classification, ab initio reconstruction, and three rounds of heterogenous refinement, a 5 Å density map was reconstituted containing 397,982 particles, which were re-extracted with pixel size of 1.08 Å. The reconstituted particles were classified by 3D classification in principal component analysis (PCA) mode. After non-uniform refinement and local refinement, a 3.27 Å density map was generated from 115,221 particles (Supplementary Fig. S2a).

For hOCT1–spironolactone complexes in LMNG micelles, data processing was performed similarly to the DDM sample, apart from retainment of the preferred orientation particles by Kang (developed by Fang Kong, https://github.com/phoeningo/ksoft) after three rounds of heterogenous refinement. The final map was reconstituted from 602,445 particles and reached a resolution of 2.98 Å.

For the hOCT1–metformin sample in DDM micelles, 8,552,079 particles were extracted with a pixel size of 1.69 Å. The previously solved hOCT1-S1 and hOCT1-S2 structures were used as initial references. After four rounds of reference-guided heterogenous refinement, selected particles were subjected to 2D classification, and particles selected from the 2D classification were used to build initial models, which suggested the existence of a new outward occluded state. The initial models were used as references to redo the reference-guided 3D classification of the full set of particles. Three rounds of heterogenous refinement and one round of ab initio reconstruction resulted in 5 Å density maps of the three conformations. After non-uniform refinement and local refinement, the reconstructed density map for the outward open conformation reached 4.14 Å resolution with 342,359 particles, while the density maps for the outward and inward occluded conformations reached 3.98 Å and 3.77 Å resolution with 194,351 particles and 118,673 particles, respectively.

For the hOCT1–Nb5660 sample, the data processing procedure was performed similarly to that of the hOCT1-spironolactone sample in LMNG micelles. After 3D classification, non-uniform refinement and local refinement, the selected 654,306 particles resulted in a 3.02 Å density map. The 3D Var and 3D Var display movie inspired the re-classification of particles. TM mask was added to do the non-align 3D classification using RELION 3.2^50^. Density maps with different conformations in TM4 and TM11 (corresponding to partially open 1, 2 and fully open conformations) were reconstructed to 3.23, 3.28, and 3.26 Å, with 170,244, 110,551, and 132,432 particles, respectively.

### Model building and refinement

The initial models of hOCT1 and Nb56 were generated using AlphaFold2^21^. These models were docked into the density maps by Chimera^51^, then adjusted manually and refined using COOT^52^. The coordinates and restraint files of spironolactone and metformin were generated by PHENIX with SMILES strings as input^53^. Subsequently, the models were automatically refined in PHENIX real_space_refine and manually adjusted in COOT for several iterations. The final validation statistics including Ramachandran plots, Clashscore were calculated with Phenix and MolProbity (Supplementary·Table·S1). The structure figures were prepared using PyMOL (The PyMOL Molecular Graphics System, Version 1.3, Schrödinger, LLC.) and ChimeraX^54^.

### ^3^H-metformin and ^3^H-MPP^+^ uptake

Wild-type hOCT1 with C terminal GFP tag was cloned into the pLJM1 vector. The lentivirus was generated in HEK293T cells by co-transfecting pLJM1-hOCT1, psPAX2 and pMD2.G in a ratio of 5:2:3. After 60 h of incubation, the resulting lentivirus was used to infect HEK293T cells. A stable expression hOCT1 cell line was selected using DMEM–10% fetal bovine serum (FBS) with 2 μg/mL puromycin for about 48 h. The cell lines for hOCT1 mutants were generated in the same way. Expression levels were detected by a microplate reader (Perkin Elmer) with the excitation at 488 nm and emission at 509 nm (Supplementary Fig. S13).

The day before the radiotracer uptake assays, cells were seeded onto poly-D-lysine (PDL) coated 96-well plates with a cell density reaching near 100% confluence before uptake. The cells were incubated with 50 μL reaction buffer (HBSS containing 74 nM ^3^H-metformin (27 Ci/mmol) or 12 nM ^3^H-MPP^+^ (83.9 Ci/mmol)) at room temperature for 10 min. The reaction was stopped by replacing the reaction buffer with pre-cooled HBSS three times. The cells were lysed with HBSS containing 1% SDS. Lysates were transferred to a counting 96-well plate with 200 μL scintillating agent in each well. Counts were read on a Microbeta2 plate reader (Perkin Elmer).

### Fluorescence substrate ASP^+^ uptake and inhibition assays

Cell lines stably expressing hOCT1 or hOCT1 mutants were seeded into black 96-well plate in the same density. When the cell density reached near 100% confluence the next day, the medium was replaced by HBSS with varying concentrations of ASP^+^. After 5 min, the reaction was stopped by replacing the reaction buffer with pre-cooled HBSS three times. Lysates were generated as described above, and the counts were read on a microplate reader (Perkin Elmer) with the excitation at 473 nm and emission at 610 nm. The cell densities of hOCT1 or hOCT1 mutants were confirmed by measuring protein concentration using a BCA kit (Beyotime Biotechnology).

For the ASP^+^ uptake inhibition assay, cells were preincubated with 50 μL HBSS containing varied concentrations of inhibitor for 20 min. Afterwards, HBSS containing 5 μM ASP^+^ was added to the wells. The cells were washed with pre-cooled HBSS three times. Counts were recorded as described above. The *K*_m_, *V*_max_, and IC_50_ were calculated using GraphPad Prism, through fitting normalized data with non-liner regression.

### Supplementary information

Supplementary Fig. S1 Biochemical characterization of hOCT1.

Supplementary Fig. S2 Cryo-EM data processing of hOCT1-spironolactone (hOCT1-S1) in DDM.

Supplementary Fig. S3 Cryo-EM data processing of hOCT1-spironolactone (hOCT1-S2) samples in LMNG.

Supplementary Fig. S4 Cryo-EM data processing of hOCT1-metformin (hOCT1-M) complexes.

Supplementary Fig. S5 Cryo-EM data processing of hOCT1-Nb5660 (hOCT1-apo) complex.

Supplementary Fig. S6 Substrate recognition by hOCT1.

Supplementary Fig. S7 Conformation rearrangement and gating of hOCT1.

Supplementary Fig. S8 Cation-π/π-π interactions between OCT and its substrates.

Supplementary Fig. S9 YER motif in SLC22 family.

Supplementary Fig. S10 Spironolactone binding of hOCT1.

Supplementary Fig. S11 Inhibition of hOCTs by steroid hormones or analogs.

Supplementary Fig. S12 A potential substrate entrance pathway.

Supplementary Fig. S13 Expression levels of hOCT1 WT and mutants.

Supplementary Table S1. Cryo-EM data collection, refinement, and validation statistics

## Data Availability

The cryo-EM maps have been deposited into the Electron Microscopy Data Bank under accession numbers EMD-36651, EMD-36652, EMD-36653, EMD-36654, EMD-36655, EMD-36656, EMD-36657, EMD-36658. The coordinates have been deposited into the Protein Data Bank under accession numbers 8JTS, 8JTT, 8JTV, 8JTW, 8JTX, 8JTY, 8JTZ, 8JU0. Correspondence and requests for materials should be addressed to C.Y., L. Chen, and X.L.
