# Supplementary material for: Structural insights into human organic cation transporter 1 transport and inhibition
Source: Cell Discov. 2024 Mar 15;10:30. doi: 10.1038/s41421-024-00664-1 (PMC10940649; doi:10.1038/s41421-024-00664-1)
Supplement: Supplementary file 1 — Supplementary Fig. S1 Biochemical characterization of hOCT1. [file 41421_2024_664_MOESM1_ESM.pdf]

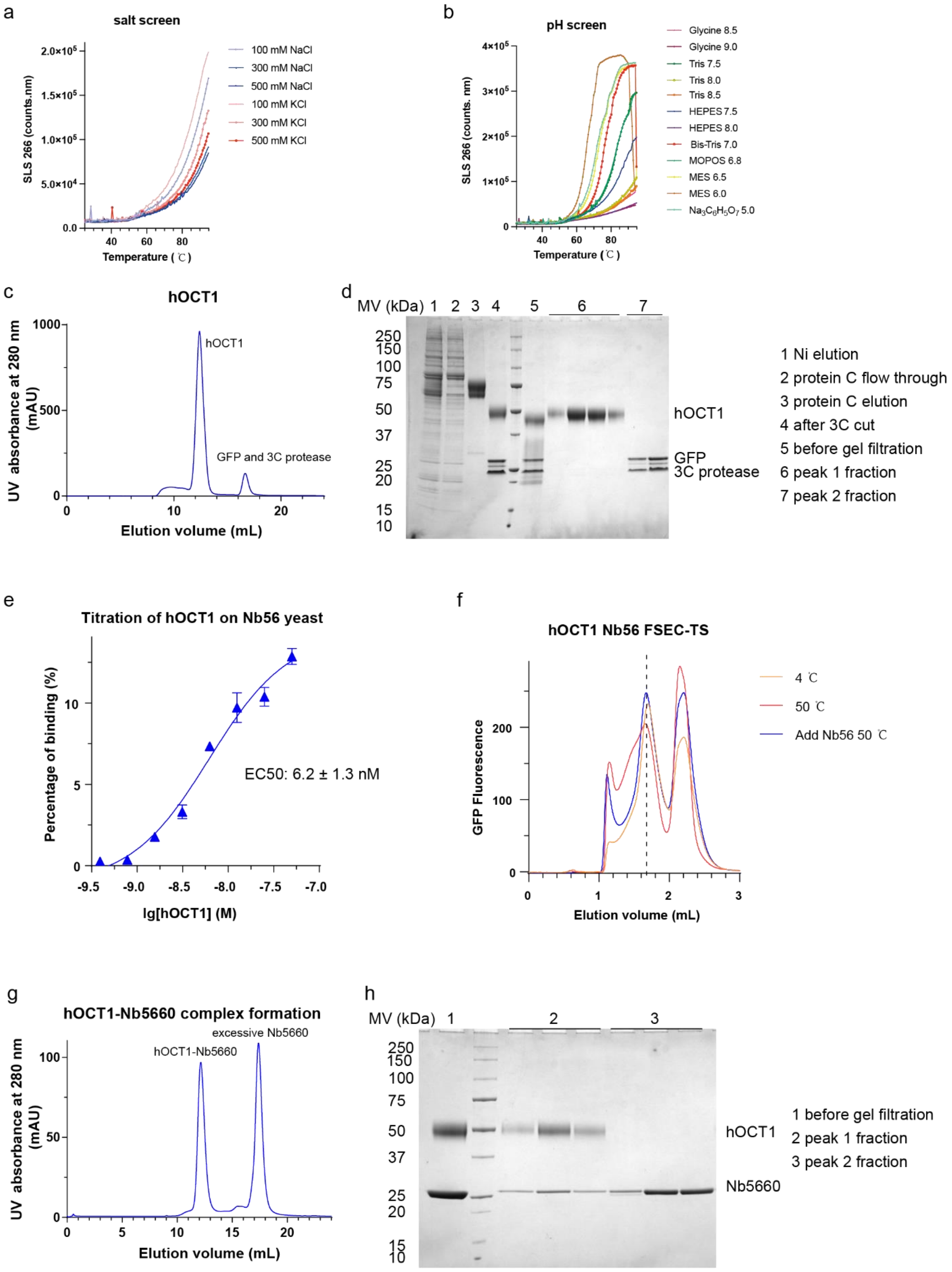

Supplementary Fig. S1 Biochemical characterization of hOCT1.

a, Protein aggregation profiles of hOCT1 in different salt concentrations at various temperatures.

b, Protein aggregation profiles of hOCT1 at different pH and at various temperatures.

c, The representative size exclusion chromatography profile of purified hOCT1.

d, A representative SDS-PAGE gel of purified hOCT1 protein.

e, On-yeast titration of Nb56-expressing yeast with various concentration of hOCT1.

f, FSEC profiles of the hOCT1-Nb56 complex compared with hOCT1 alone at 4 °C and 50 °C.

g, Representative size exclusion chromatography profile of the purified hOCT1-Nb5660 complex.

h, Representative SDS-PAGE gel of the purified hOCT1-Nb5660 complex.
