# Supplementary material for: Structural insights into human organic cation transporter 1 transport and inhibition
Source: Cell Discov. 2024 Mar 15;10:30. doi: 10.1038/s41421-024-00664-1 (PMC10940649; doi:10.1038/s41421-024-00664-1)
Supplement: Supplementary file 2 — Supplementary Fig. S2 Cryo-EM data processing of hOCT1-spironolactone (hOCT1-S1) in DDM. [file 41421_2024_664_MOESM2_ESM.pdf]

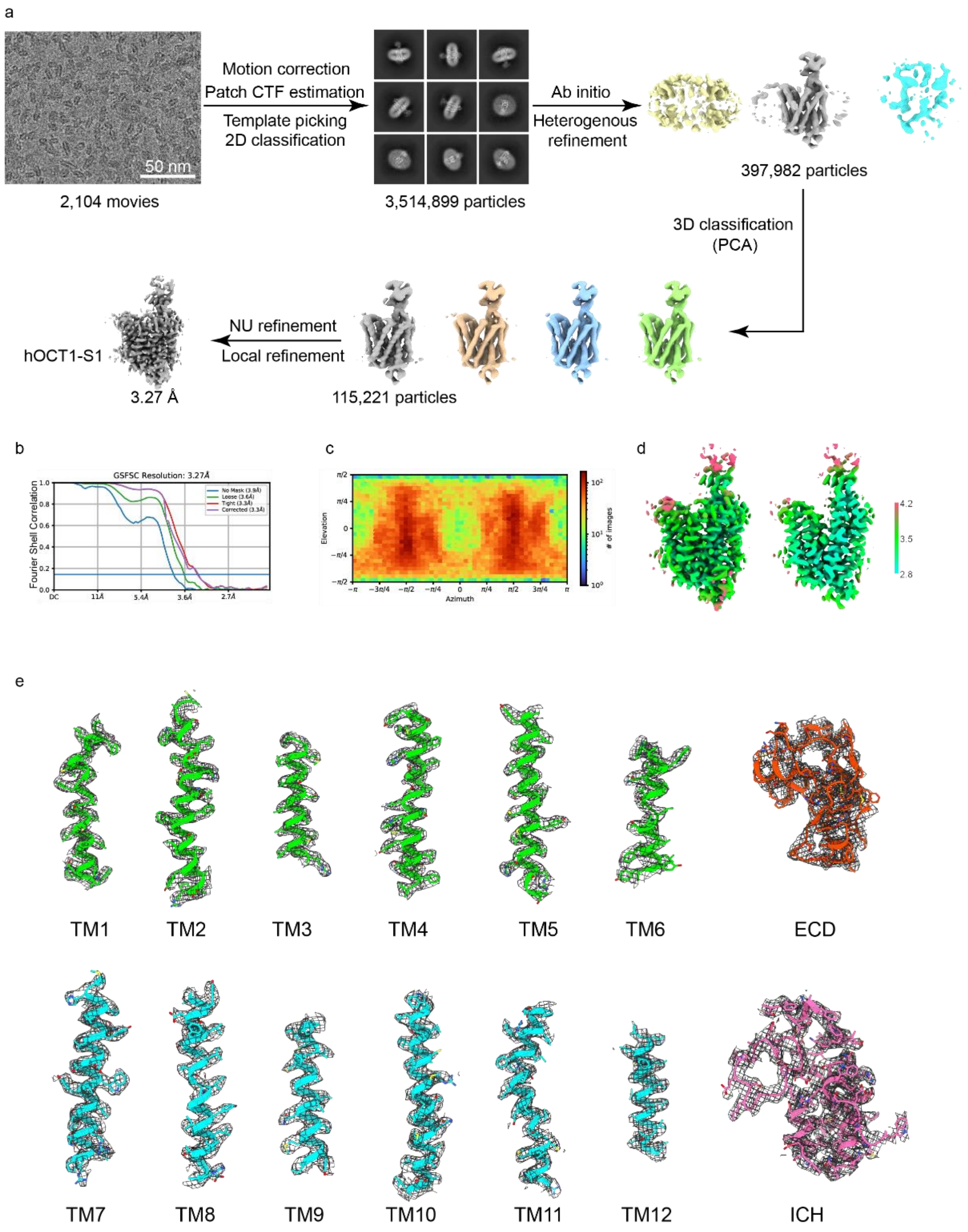

Supplementary Fig. S2 Cryo-EM data processing of hOCT1-spironolactone (hOCT1-S1) in DDM.

a, Workflow of the data processing pipeline for the hOCT1-spironolactone samples in DDM.

b, Gold-standard FSC curves of the refinement of hOCT1-spironolactone structures in DDM micelles.

c, Angular distribution of the particles used for the final reconstruction.

d, The local resolution of the hOCT1-spironolactone complex in a DDM micelle.

e, Cryo-EM density maps of the transmembrane helices, ECD, and ICHD.
