# Supplementary material for: Structural insights into human organic cation transporter 1 transport and inhibition
Source: Cell Discov. 2024 Mar 15;10:30. doi: 10.1038/s41421-024-00664-1 (PMC10940649; doi:10.1038/s41421-024-00664-1)
Supplement: Supplementary file 4 — Supplementary Fig. S4 Cryo-EM data processing of hOCT1-metformin (hOCT1-M) complexes. [file 41421_2024_664_MOESM4_ESM.pdf]

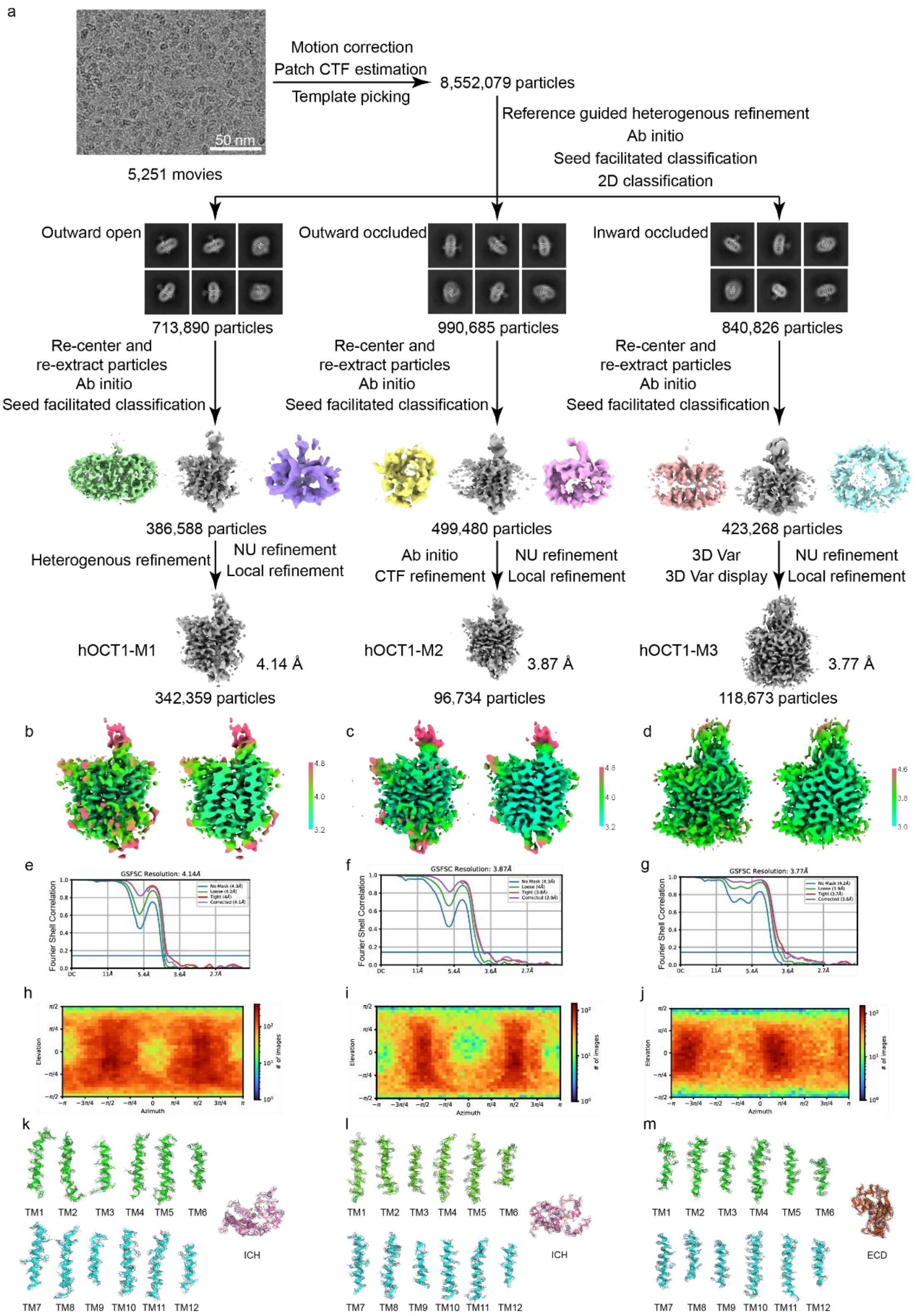

Supplementary Fig. S4 Cryo-EM data processing of hOCT1-metformin (hOCT1-M) complexes.

a, Data processing pipeline for hOCT1-metformin structures.

b-d, Local resolution of hOCT1-metformin structures in the outward open (b), outward occluded (c), and inward occluded (d) conformations.

e-g, Gold-standard FSC curves of the hOCT1-metformin complexes in the outward open (e), outward occluded (f), and inward occluded (g) conformations.

h-j, The angular distribution of particles for the final reconstruction of the hOCT1-metformin complex in the outward open (h), outward occluded (i), and inward occluded (j) conformations.

k-m, Cryo-EM density maps of hOCT1-metformin structures in outward open (k), outward occluded (l), and inward occluded (m) conformations.
