# Supplementary material for: Structural insights into human organic cation transporter 1 transport and inhibition
Source: Cell Discov. 2024 Mar 15;10:30. doi: 10.1038/s41421-024-00664-1 (PMC10940649; doi:10.1038/s41421-024-00664-1)
Supplement: Supplementary file 5 — Supplementary Fig. S5 Cryo-EM data processing of hOCT1-Nb5660 (hOCT1-apo) complex. [file 41421_2024_664_MOESM5_ESM.pdf]

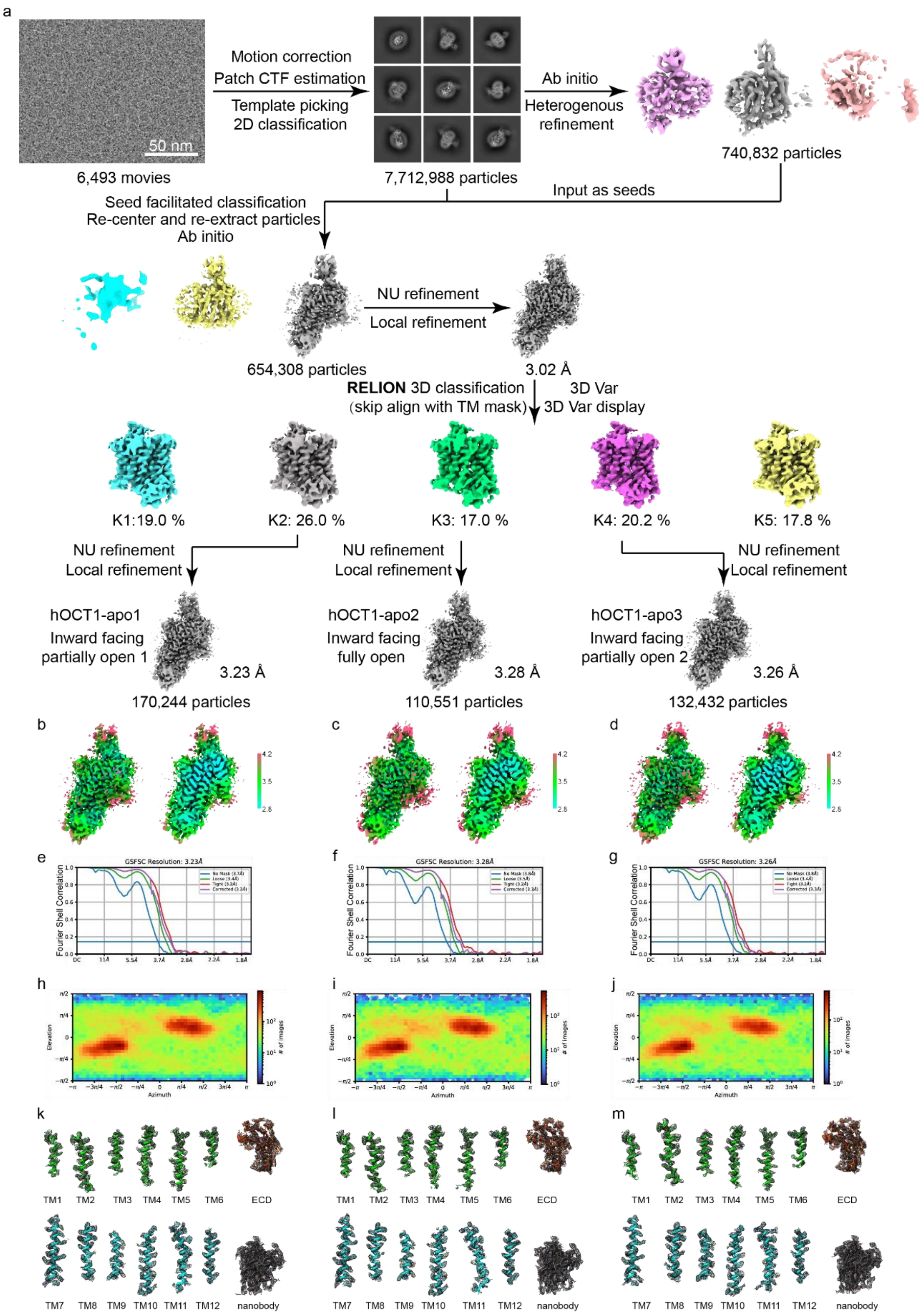

Supplementary Fig. S5 Cryo-EM data processing of hOCT1-Nb5660 (hOCT1-apo) complex.

a, Data process pipeline for hOCT1-Nb5660 structures.

b-d, Local resolution of hOCT1-Nb5660 structures in the inward facing partially open 1 (b), partially open 2 (d), and fully open (c) conformations.

e-g, Gold-standard FSC curves of hOCT1-Nb5660 structures in inward facing partially open 1 (e), partially open 2 (g), and fully open (f) conformations.

h-j, The angular distribution of particles for the final reconstruction of hOCT1-Nb5660 complexes in inward facing partially open 1 (h), partially open 2 (j), and fully open (i) conformations.

k-m, Cryo-EM density maps of hOCT1-Nb5660 structures in the inward facing partially open 1 (k), partially open 2 (m), and fully open (l) conformations.
