# Supplementary material for: Structural insights into human organic cation transporter 1 transport and inhibition
Source: Cell Discov. 2024 Mar 15;10:30. doi: 10.1038/s41421-024-00664-1 (PMC10940649; doi:10.1038/s41421-024-00664-1)
Supplement: Supplementary file 6 — Supplementary Fig. S6 Substrate recognition by hOCT1. [file 41421_2024_664_MOESM6_ESM.pdf]

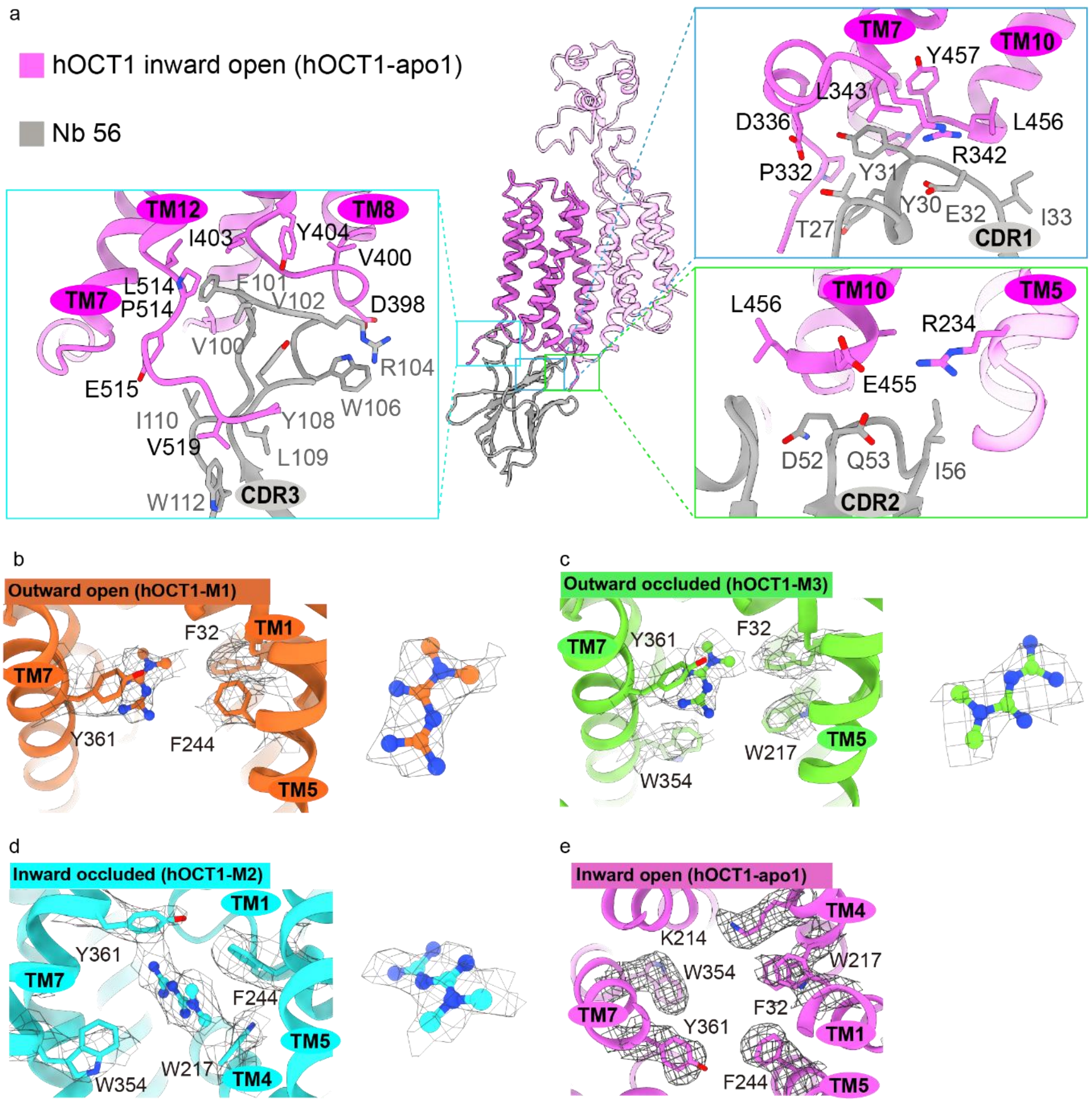

Supplementary Fig. S6 Substrate recognition by hOCT1.

a, Structure of the hOCT1-Nb5660 complex in an inward open conformation with close-up view of the complementarity-determining region 1 (CDR1) interacting with intracellular loop 3 (ICL3) and ICL5 (top right), CDR2 interacting with TM5 and TM10 (bottom right), and CDR3 interacting with ICL4 and the C terminus (C-ter) (bottom left).

b-e, Electron densities of metformin and the binding pocket residues in outward open (hOCT1-M1) (b), outward occluded (hOCT1-M2) (c), inward occluded (hOCT1-M3) (d), and inward open (hOCT1-apo1) (e) conformations.
