# Supplementary material for: Structural insights into human organic cation transporter 1 transport and inhibition
Source: Cell Discov. 2024 Mar 15;10:30. doi: 10.1038/s41421-024-00664-1 (PMC10940649; doi:10.1038/s41421-024-00664-1)
Supplement: Supplementary file 7 — Supplementary Fig. S7 Conformation rearrangement and gating of hOCT1. [file 41421_2024_664_MOESM7_ESM.pdf]

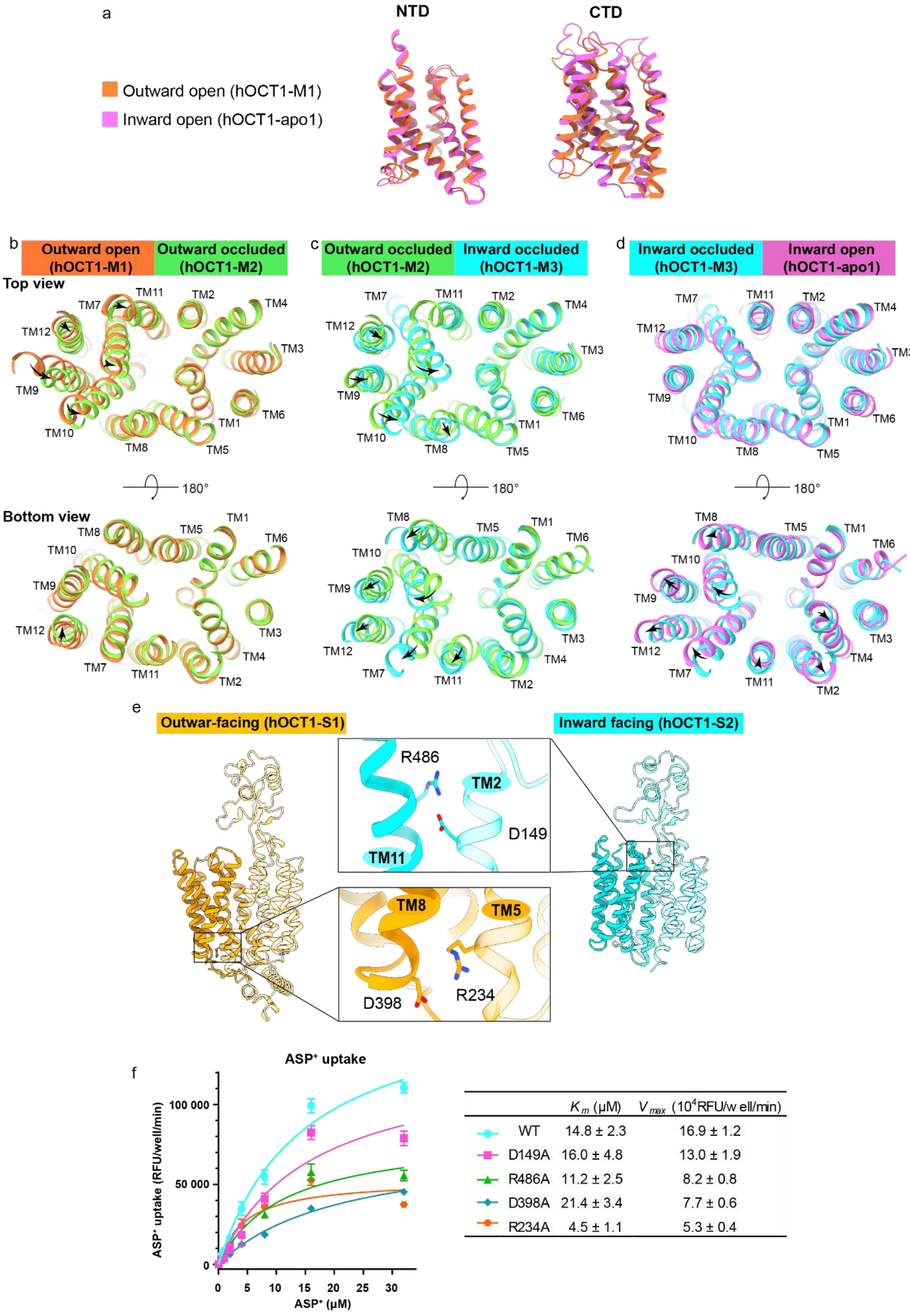

	K_m (μM)	V_{max} (10^4RFU/w ell/min)
WT	14.8 ± 2.3	16.9 ± 1.2
D149A	16.0 ± 4.8	13.0 ± 1.9
R486A	11.2 ± 2.5	8.2 ± 0.8
D398A	21.4 ± 3.4	7.7 ± 0.6
R234A	4.5 ± 1.1	5.3 ± 0.4

Supplementary Fig. S7 Conformation rearrangement and gating of hOCT1.

a, Structural comparison of the NTD and CTD domains in outward open (hOCT1-M1) (orange) and inward open (hOCT1-apo1) (purple) conformations.

b, Top and bottom views of hOCT1 in outward open (hOCT1-M1) (orange) and outward occluded (hOCT1-M2) (green) conformations. The extracellular ends of transmembrane helices exhibit conformational rearrangement.

c, Top and bottom views of hOCT1 in outward occluded (hOCT1-M2) (green) and inward occluded (hOCT1-M3) (cyan) conformations. Both the extracellular ends and the intracellular ends of transmembrane helices in CTD exhibit conformational rearrangement.

d, Top and bottom views of hOCT1 in inward occluded (hOCT1-M3) (cyan) and inward open (hOCT1-apo1) (magenta) conformations. The intracellular ends of transmembrane helices in CTD exhibit conformational rearrangement.

e, Overall structure of outward facing (hOCT1-S1) (orange) and inward facing (hOCT1-S2) (cyan) hOCT1 with close-up view of the inward and outward gates.

f, Transport activities of inward and outward gates mutations at various ASP^+ concentrations. Data are shown as mean ± SEM of 3 independent experiments.
