# Supplementary material for: Structural insights into human organic cation transporter 1 transport and inhibition
Source: Cell Discov. 2024 Mar 15;10:30. doi: 10.1038/s41421-024-00664-1 (PMC10940649; doi:10.1038/s41421-024-00664-1)
Supplement: Supplementary file 8 — Supplementary Fig. S8 Cation-π/π-π interactions between OCT and its substrates. [file 41421_2024_664_MOESM8_ESM.pdf]

a

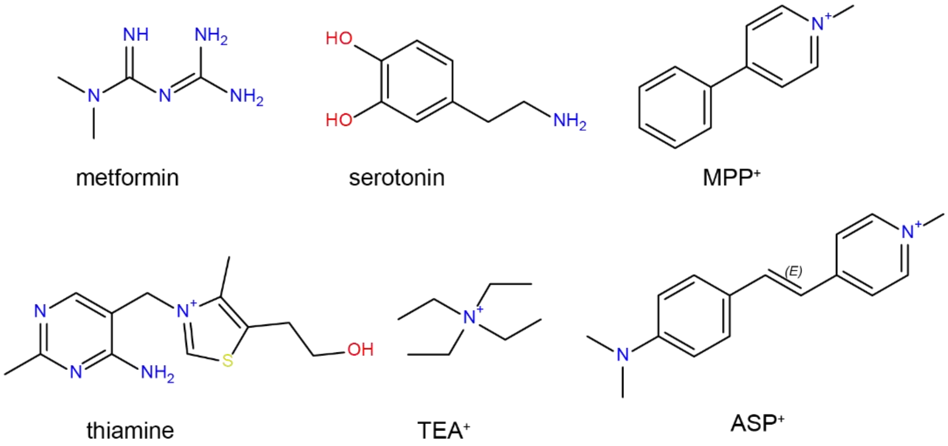

b

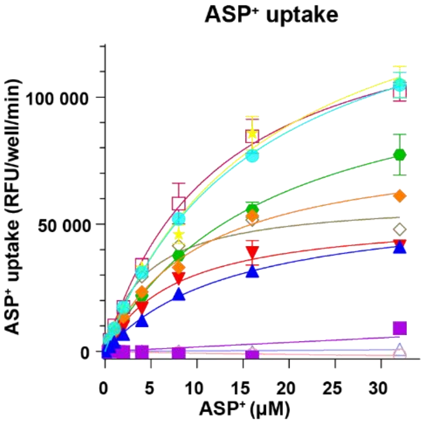

	K_m (μM)	V_{max} (10^4RFU/well/min)
WT	16.4 ± 1.2	15.8 ± 0.6
F32A	14.1 ± 1.3	6.0 ± 2.5
K214A	/	/
W217A	10.3 ± 0.8	8.2 ± 2.6
Q241A	7.8 ± 1.0	5.4 ± 0.2
F244A	17.6 ± 1.7	11.9 ± 0.6
W354A	/	/
D357A	17.4 ± 1.8	16.6 ± 0.8
Y361A	/	/
Y361F	12.3 ± 1.2	14.4 ± 0.6
Q362A	4.3 ± 0.5	6.0 ± 0.2

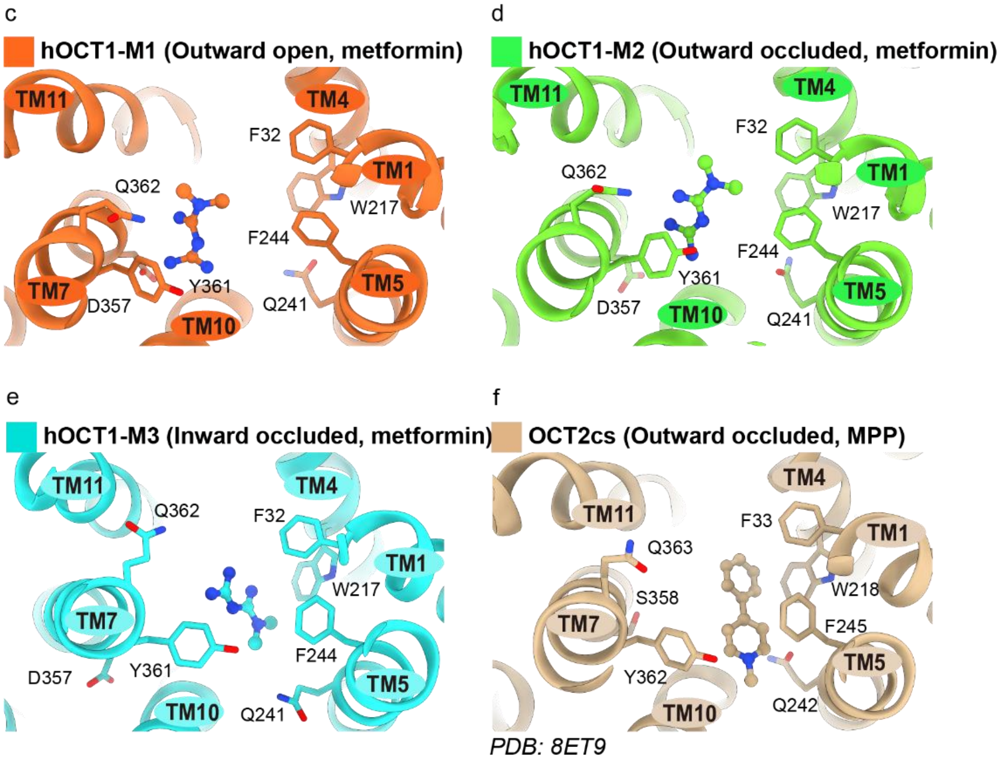

Supplementary Fig. S8 Cation-π/π-π interactions between OCT and its substrates.

a, Chemical structures of the hOCT1 substrates metformin, thiamine, serotonin, MPP^+, and ASP^+.

b, Transport activities of hOCT1 with mutations in metformin binding pocket residues at various ASP^+ concentrations. Data are shown as mean ± SEM of 3 independent experiments.

c-e, Cation-π interaction between metformin and hOCT1 in hOCT1-M1 (c), hOCT1-M2 (d), and hOCT1-M3 (e).

f, π-π interaction between MPP^+ and OCT2cs.
