# Supplementary material for: Structural insights into human organic cation transporter 1 transport and inhibition
Source: Cell Discov. 2024 Mar 15;10:30. doi: 10.1038/s41421-024-00664-1 (PMC10940649; doi:10.1038/s41421-024-00664-1)
Supplement: Supplementary file 9 — Supplementary Fig. S9 YER motif in SLC22 family. [file 41421_2024_664_MOESM9_ESM.pdf]

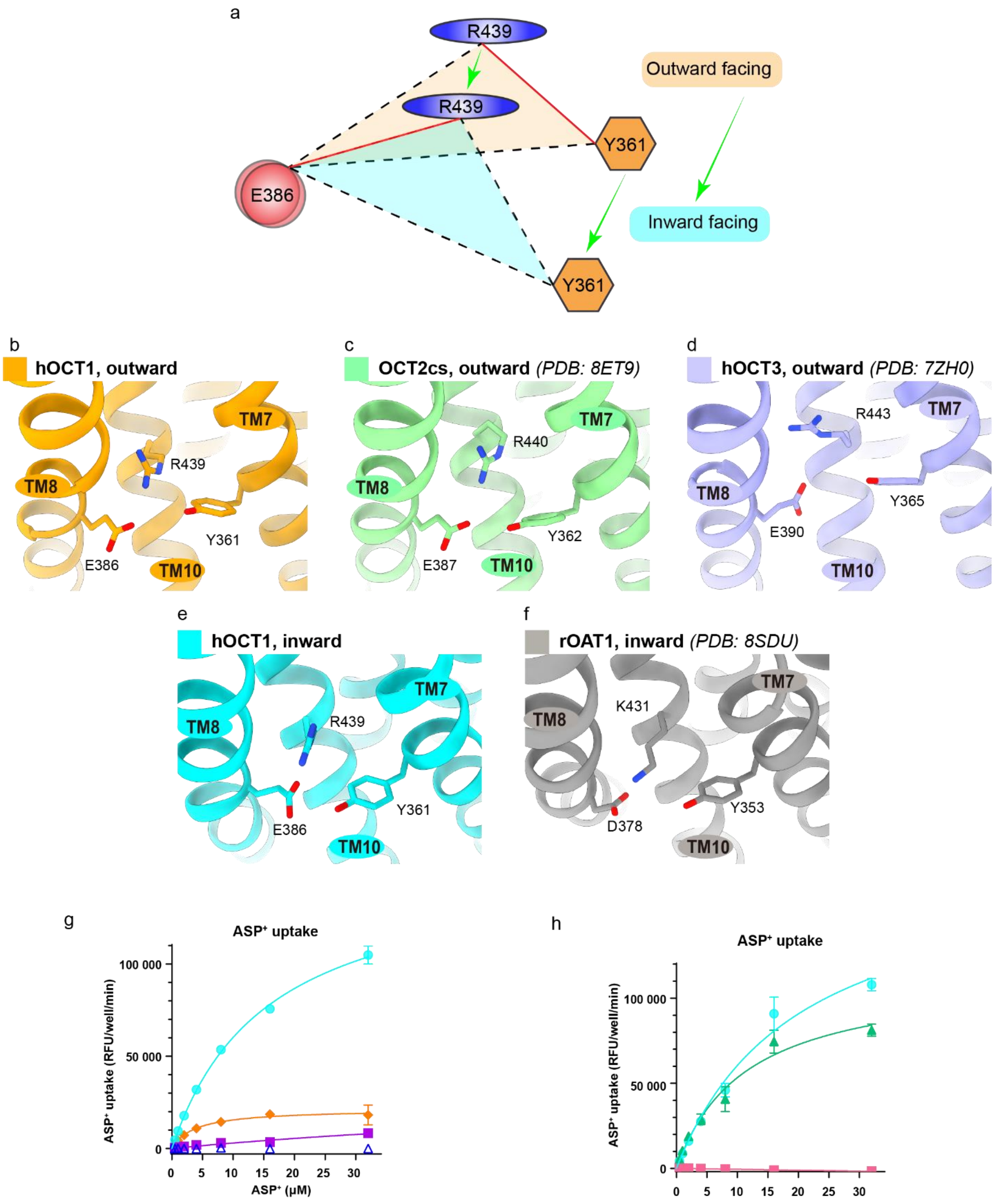

	K_m (μM)	V_{max} (10^4RFU/w ell/min)
WT	15.7 ± 1.3	15.5 ± 0.6
Y361A	/	/
E386A	>100	3.6 ± 2.1
R439A	3.9 ± 1.1	2.1 ± 0.2

	K_m (μM)	V_{max} (10^4RFU/w ell/min)
WT	20.1 ± 3.8	18.2 ± 1.8
W354A	/	/
N453A	11.4 ± 2.3	11.4 ± 1.0

Supplementary Fig. S9 YER motif in SLC22 family.

a, Schematic representation of the movement of the YER motif between outward and inward facing conformations. The red dash lines represent the key interaction in outward or inward facing conformations. The green arrows represent the moving direction.

b-f, A sliced view of the YER motif in hOCT1-S1 (b), OCT2cs (c), hOCT3 (d), hOCT1-S2 (e), and rOAT1 (f).

g, Mutating any residue of the YER motif impaired ASP^+ transport activity by hOCT1. Data are shown as mean ± SEM of 3 independent experiments.

h, Transport activities of W354A and N453A mutations at various ASP^+ concentrations. Data are shown as mean ± SEM of 3 independent experiments.
