# Supplementary material for: Structural insights into human organic cation transporter 1 transport and inhibition
Source: Cell Discov. 2024 Mar 15;10:30. doi: 10.1038/s41421-024-00664-1 (PMC10940649; doi:10.1038/s41421-024-00664-1)
Supplement: Supplementary file 10 — Supplementary Fig. S10 Spironolactone binding of hOCT1. [file 41421_2024_664_MOESM10_ESM.pdf]

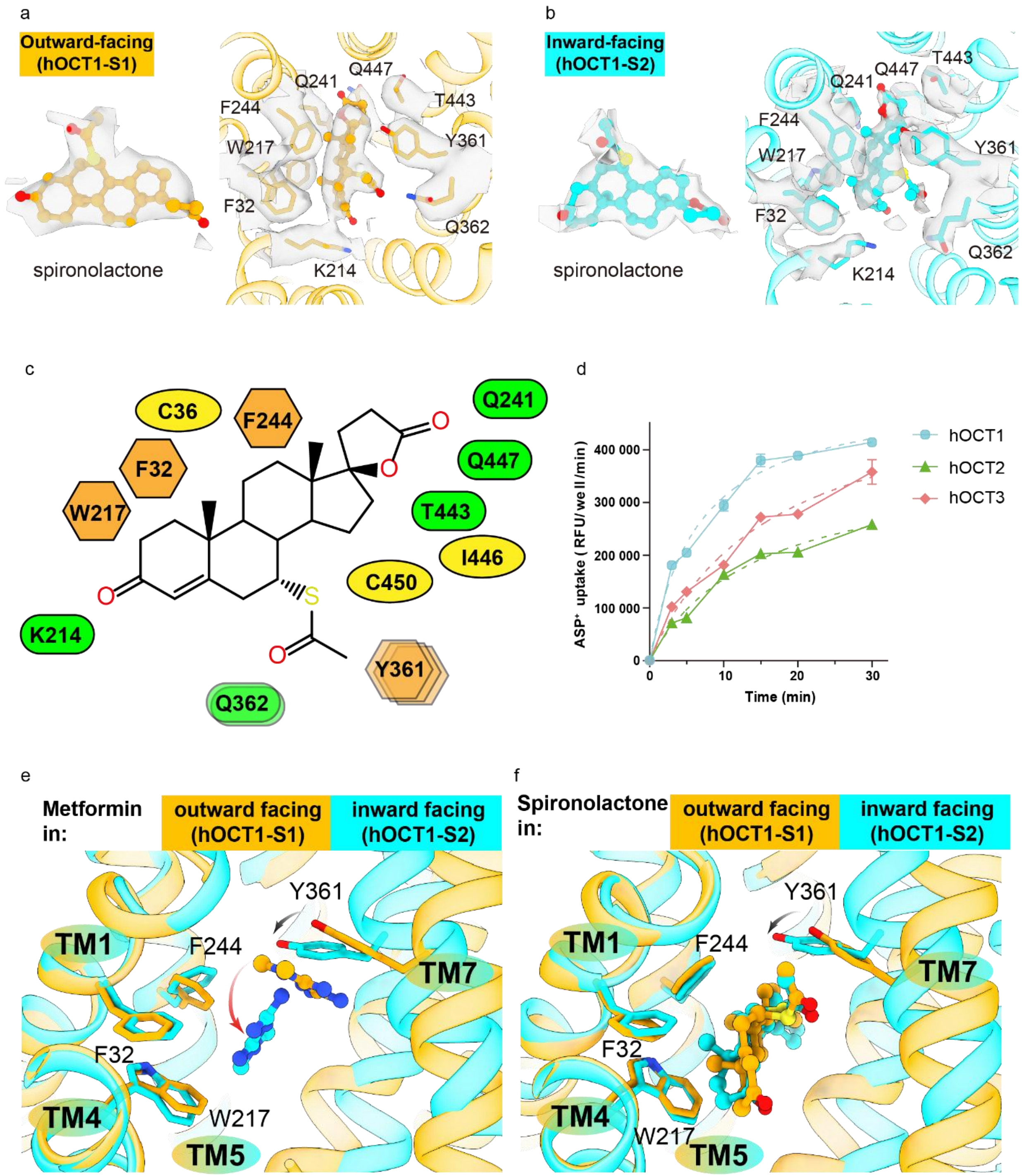

Supplementary Fig. S10 Spironolactone binding of hOCT1.

a-b, Electron densities of spironolactone and binding pocket residues in outward facing (hOCT1-S1) (a), inward facing (hOCT1-S2) (b) conformations.

c, Schematic representation of the spironolactone binding site. The hydrophilic residues are colored in green, the aromatic residues are colored in orange and shown as hexagon, while the hydrophobic residues that are different

in the hOCT1, hOCT2, and hOCT3 are colored in yellow and shown as oval.

d, Time course of ASP^+ uptake in HEK293T cells expressing hOCT1, hOCT2, or hOCT3. Data are shown as mean ± SEM of 3 independent experiments.

e-f, Structural comparison of metformin (e) or spironolactone (f) binding pockets in outward facing (left: hOCT1-M1, right: hOCT1-S1) (orange) and inward facing (left: hOCT1-M3, right: hOCT1-S2) (cyan) conformations.
