# Supplementary material for: Structural insights into human organic cation transporter 1 transport and inhibition
Source: Cell Discov. 2024 Mar 15;10:30. doi: 10.1038/s41421-024-00664-1 (PMC10940649; doi:10.1038/s41421-024-00664-1)
Supplement: Supplementary file 11 — Supplementary Fig. S11 Inhibition of hOCTs by steroid hormones or analogs. [file 41421_2024_664_MOESM11_ESM.pdf]

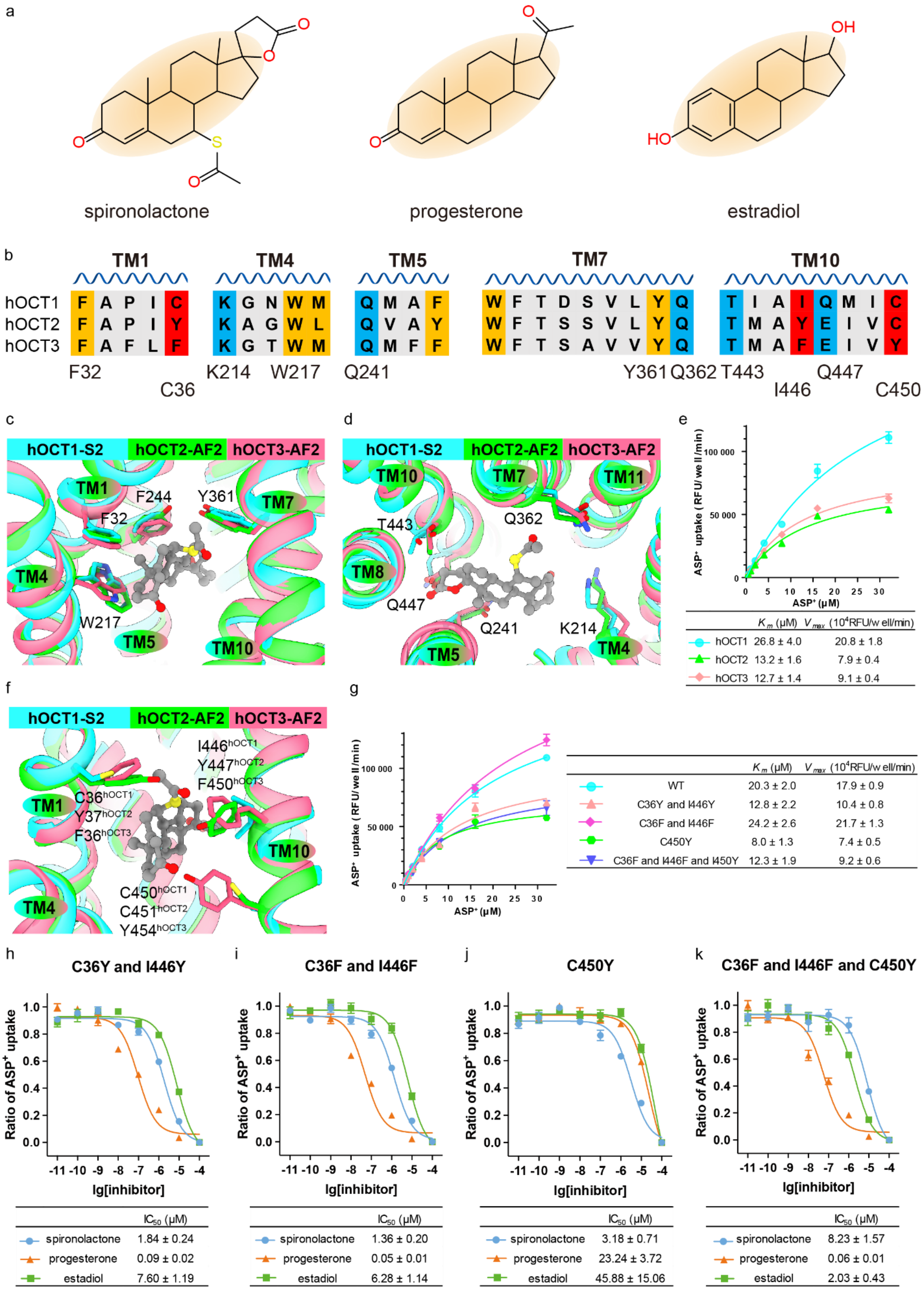
a
spironolactone
progesterone
estradiol
b
TM1
TM4
TM5
TM7
TM10
hOCT1 F A P I C K G N W M Q M A F W F T D S V L Y Q T I A I Q M I C
hOCT2 F A P I Y K A G W L Q V A Y W F T S S V L Y Q T M A Y E I V C
hOCT3 F A F L F K G T W M Q M F F W F T S A V V Y Q T M A F E I V Y
F32 C36 K214 W217 Q241 Y361 Q362 T443 I446 Q447 C450
c
hOCT1-S2 hOCT2-AF2 hOCT3-AF2
TM1 F244 Y361 TM7 F32 TM4 W217 TM5 TM10
d
hOCT1-S2 hOCT2-AF2 hOCT3-AF2
TM10 TM7 TM11 T443 Q362 TM8 Q447 Q241 K214 TM5 TM4
e
ASP⁺ uptake (RFU/ well/min)
ASP⁺ (μM)
Km (μM) Vmax (10⁴RFU/well/min)
hOCT1 26.8 ± 4.0 20.8 ± 1.8
hOCT2 13.2 ± 1.6 7.9 ± 0.4
hOCT3 12.7 ± 1.4 9.1 ± 0.4
f
hOCT1-S2 hOCT2-AF2 hOCT3-AF2
I446hOCT1 Y447hOCT2 F450hOCT3
TM1 C36hOCT1 Y37hOCT2 F36hOCT3
TM10
C450hOCT1 C451hOCT2 Y454hOCT3
TM4
g
ASP⁺ uptake (RFU/ well/min)
ASP⁺ (μM)
Km (μM) Vmax (10⁴RFU/well/min)
WT 20.3 ± 2.0 17.9 ± 0.9
C36Y and I446Y 12.8 ± 2.2 10.4 ± 0.8
C36F and I446F 24.2 ± 2.6 21.7 ± 1.3
C450Y 8.0 ± 1.3 7.4 ± 0.5
C36F and I446F and I450Y 12.3 ± 1.9 9.2 ± 0.6
h
C36Y and I446Y
Ratio of ASP⁺ uptake
lg[inhibitor]
IC50 (μM)
spironolactone 1.84 ± 0.24
progesterone 0.09 ± 0.02
estadiol 7.60 ± 1.19
i
C36F and I446F
Ratio of ASP⁺ uptake
lg[inhibitor]
IC50 (μM)
spironolactone 1.36 ± 0.20
progesterone 0.05 ± 0.01
estadiol 6.28 ± 1.14
j
C450Y
Ratio of ASP⁺ uptake
lg[inhibitor]
IC50 (μM)
spironolactone 3.18 ± 0.71
progesterone 23.24 ± 3.72
estadiol 45.88 ± 15.06
k
C36F and I446F and C450Y
Ratio of ASP⁺ uptake
lg[inhibitor]
IC50 (μM)
spironolactone 8.23 ± 1.57
progesterone 0.06 ± 0.01
estadiol 2.03 ± 0.43

Supplementary Fig. S11 Inhibition of hOCTs by steroid hormones or analogs.

a, The chemical structure of steroid hormones or analogs used in this study, including spironolactone, progesterone, and estradiol.

b, Sequence alignment of the spironolactone binding pocket residues in hOCT1, hOCT2 and hOCT3. The hydrophilic residues are colored in blue, and hydrophobic residues are colored in orange. Residues with distinct properties (Cys or Ile in hOCT1 vs aromatic residues in hOCT2 or hOCT3) are highlighted in red.

c-d, Structural comparison of the spironolactone binding pocket between inward facing hOCT1 (hOCT1-S2) (cyan), AlphaFold2 predicted hOCT2 (lime), and AlphaFold2 predicted hOCT3 (hot pink). Hydrophobic residues are shown as sticks in (c), while hydrophilic residues are shown as sticks in (d).

e, ASP^+ transport activities of hOCT1, hOCT2, and hOCT3. Data are shown as mean ± SEM of 3 independent experiments.

f, Structural comparison of the spironolactone binding pocket between inward facing (hOCT1-S2) hOCT1 (cyan), AlphaFold2 predicted hOCT2 (lime), and AlphaFold2 predicted hOCT3 (hot pink). Different residues are shown as sticks.

g, ASP^+ transport activities of WT or mutated hOCT1 with mutations that mimic the spironolactone binding pocket of hOCT2 or hOCT3. Data are shown as mean ± SEM of 3 independent experiments.

h-k, Inhibition of ASP^+ uptake by spironolactone (cyan), progesterone (orange), and estradiol (green) in HEK293T cells stably expressing hOCT1 with C36Y and I446Y (h), C36F and I446F (i), C450Y (j), and C36F and I446F and C450Y (k) mutations. Data are shown as mean ± SEM of 3 independent experiments.
