# Supplementary material for: Structural insights into human organic cation transporter 1 transport and inhibition
Source: Cell Discov. 2024 Mar 15;10:30. doi: 10.1038/s41421-024-00664-1 (PMC10940649; doi:10.1038/s41421-024-00664-1)
Supplement: Supplementary file 12 — Supplementary Fig. S12 A potential substrate entrance pathway. [file 41421_2024_664_MOESM12_ESM.pdf]

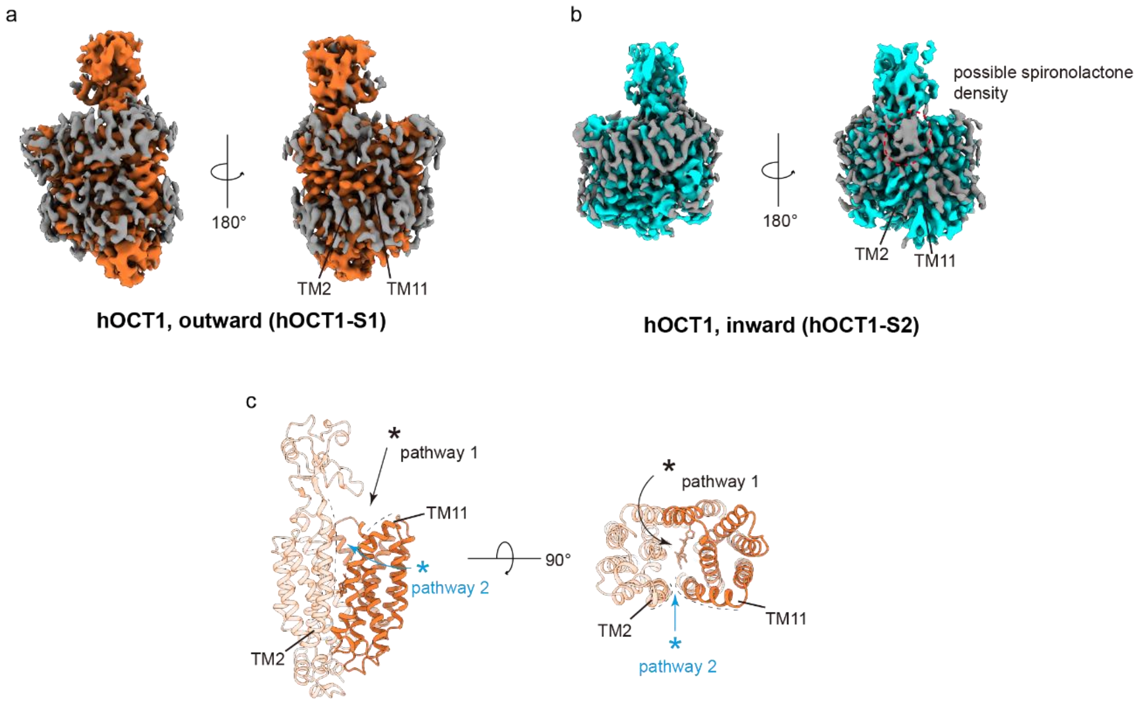

Supplementary Fig. S12 A potential substrate entrance pathway.

a-b, The cryo-EM map of hOCT1-S1 (a) and hOCT1-S2 (b) reveal densities that may correspond to ordered lipids surrounding the transmembrane helices of hOCT1. The red dashed circle highlights a blob with a different shape, potentially from spironolactone, near TM2 and TM11.

c, Potential substrate/inhibitor (labeled by *) entrance pathways of hOCT1.
