# Supplementary material for: Structural insights into human organic cation transporter 1 transport and inhibition
Source: Cell Discov. 2024 Mar 15;10:30. doi: 10.1038/s41421-024-00664-1 (PMC10940649; doi:10.1038/s41421-024-00664-1)
Supplement: Supplementary file 13 — Supplementary Fig. S13 Expression levels of hOCT1 WT and mutants. [file 41421_2024_664_MOESM13_ESM.pdf]

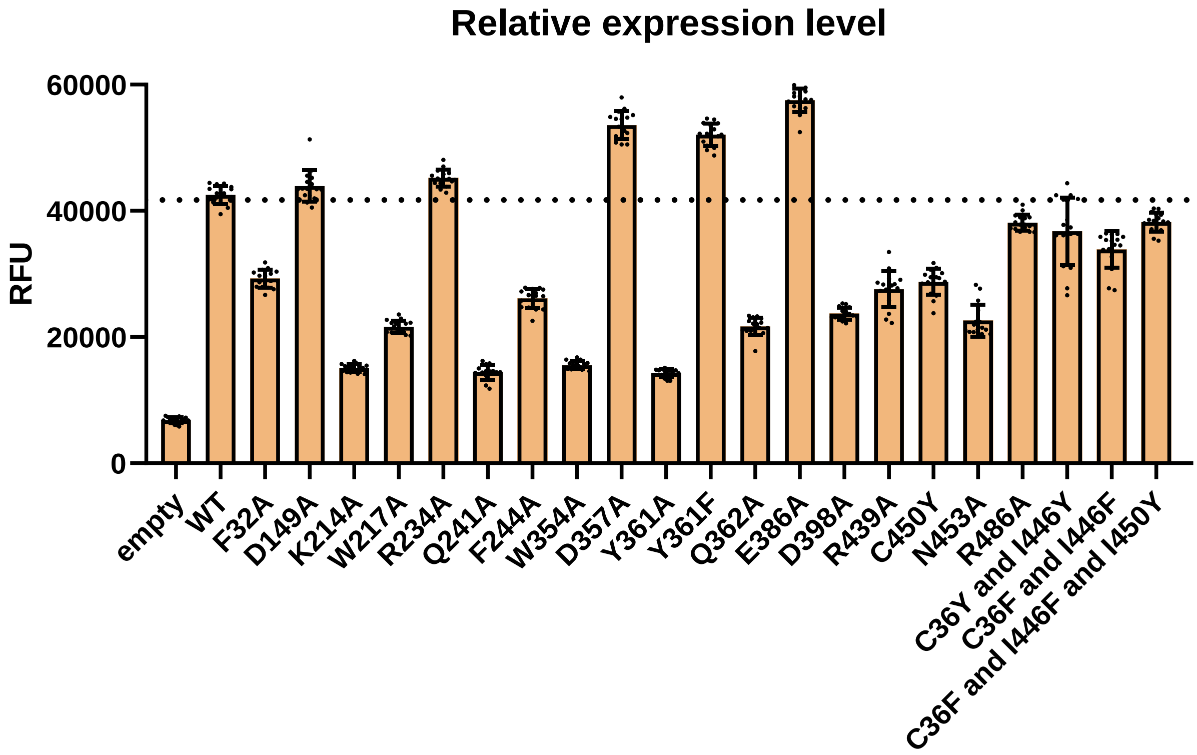


**Supplementary Fig. S13 Expression levels of hOCT1 WT and mutants.**

Expression levels of hOCT1 WT and mutant proteins. Data are shown as the mean ± SD of 4 independent experiments. The dotted line represents WT expression levels.
