# Supplementary material for: Structural insights into human organic cation transporter 1 transport and inhibition
Source: Cell Discov. 2024 Mar 15;10:30. doi: 10.1038/s41421-024-00664-1 (PMC10940649; doi:10.1038/s41421-024-00664-1)
Supplement: Supplementary file 14 — Supplementary Table S1. Cryo-EM data collection, refinement, and validation statistics [file 41421_2024_664_MOESM14_ESM.pdf]

Supplementary Table S1. Cryo-EM data collection, refinement, and validation statistics

	hOCT1-S1	hOCT1-S2	hOCT1-M1	hOCT1-M2	hOCT1-M3	hOCT1-apo1	hOCT1-apo2	hOCT1-apo3
**Data collection and processing***								
Magnification	64,000	64,000	64,000	64,000	64,000	96,000	96,000	96,000
Voltage (kV)	300	300	300	300	300	300	300	300
Electron exposure (e–/Å^2)	50	50	50	50	50	50	50	50
Defocus range (μm)	1.1-1.5	1.1-1.5	1.1-1.5	1.1-1.5	1.1-1.5	1.1-1.5	1.1-1.5	1.1-1.5
Pixel size (Å)	1.08	1.08	1.08	1.08	1.08	0.86	0.86	0.86
PDB code	8JTZ	8JU0	8JTS	8JTT	8JTV	8JTW	8JTX	8JTY
EMDB code	36657	36658	36651	36652	36653	36654	36655	36656
Symmetry imposed	C1	C1	C1	C1	C1	C1	C1	C1
Initial particle images (no.)	3,514,899	6,335,224	8,552,079	8,552,079	8,552,079	7,712,988	7,712,988	7,712,988
Final particle images (no.)	115,221	602,445	342,359	194,351	118,673	170,244	110,551	132,432
Map resolution (Å)	3.27	2.98	4.14	3.98	3.77	3.23	3.28	3.26
FSC threshold	0.143	0.143	0.143	0.143	0.143	0.143	0.143	0.143
Map resolution range (Å)	200-3.27	200-2.98	200-4.14	200-3.98	200-3.77	200-3.23	200-3.28	200-3.26
**Refinement**								
Initial model used (PDB code)	None	None	8JTZ	8JTZ	8JU0	None	8JTW	8JTW
Model resolution (Å)	3.6	3.2	4.5	4.5	4.3	3.5	3.5	3.5
FSC threshold	0.5	0.5	0.5	0.5	0.5	0.5	0.5	0.5
Model resolution range (Å)	3.2-3.6	2.9-3.2	4.0-4.5	3.1-4.5	3.4-4.3	3.1-3.5	3.1-3.5	3.1-3.5
Map sharpening *B* factor (Å^2)	-142.6	-155.7	-208.5	-179.7	-158.5	-151.7	-151.9	-156.1
Model composition								
Non-hydrogen atoms	3999	3199	3330	3321	3067	4282	4253	4274
Protein residues	530	431	461	454	437	566	566	566
Ligands	1	1	1	1	1	0	0	0
B factors (Å^2)								
Protein	104.47	64.36	77.27	32.90	16.58	71.06	89.99	77.45
Ligand	97.20	66.10	20.00	13.63	20.00	N/A	N/A	N/A
R.m.s. deviations								
Bond lengths (Å)	0.003	0.003	0.007	0.004	0.003	0.002	0.003	0.003
Bond angles (°)	0.810	0.597	0.907	0.837	0.633	0.556	0.701	0.547
Validation								
MolProbity score	1.71	1.42	1.83	1.69	1.73	1.36	1.33	1.54
Clashscore	9.71	7.60	8.91	7.52	8.10	2.70	2.73	4.12
Poor rotamers (%)	0.25	0	0.31	0	0.36	0	0.46	0
Ramachandran plot								
Favored (%)	96.78	98.35	94.97	96.00	95.82	95.71	96.07	95.00
Allowed (%)	3.22	1.65	5.03	4.00	4.18	4.29	3.93	5.00
Disallowed (%)	0	0	0	0	0	0	0	0

*The hOCT1-M1, hOCT2-M2 and hOCT1-M3 structures are from one dataset, and the hOCT1-apo1, hOCT1-apo2 and hOCT1-apo3 structures originate from a different dataset.
